# Single-cell transcriptomic profiling provides insights into the toxic effects of Zearalenone exposure on primordial follicle assembly

**DOI:** 10.7150/thno.58433

**Published:** 2021-03-05

**Authors:** Yu Tian, Ming-Yu Zhang, Ai-Hong Zhao, Li Kong, Jun-Jie Wang, Wei Shen, Lan Li

**Affiliations:** 1 College of Life Sciences, Key Laboratory of Animal Reproduction and Biotechnology in Universities of Shandong, Qingdao Agricultural University, Qingdao 266109, China.; 2 Qingdao Academy of Agricultural Sciences, Qingdao 266100, China.

**Keywords:** Zearalenone, single-cell RNA-seq, primordial follicle assembly, oocytes, granulosa cells

## Abstract

**Rationale:** Zearalenone (ZEN), a pollutant in our daily diet, seriously threatens the reproductive health of humans and animals. The primordial follicle (PF) assembly in the mouse occurs during the perinatal period, which determines the whole ovarian reserve in reproductive lifespan. In the current investigation, we administered ZEN orally to perinatal mice from 16.5 days post coitum (dpc) to postnatal day 3 (PD3), and single-cell RNA sequencing (scRNA-seq) was performed on PD0 and PD3 ovarian tissues in the offspring to check ZEN toxic to primordial follicle formation at the single cell level.

**Methods:** Ovarian tissues (*in vivo*) were examined by single cell RNA sequencing analysis, Immunostaining, and Western blotting. Ovarian tissues (*in vitro*) were examined by qRT-PCR, Immunostaining, and Western blotting.

**Results:** We found that ZEN exposure altered the developmental trajectory of both germ cells and granulosa cells. Furthermore, after establishing the cell-cell communication network between germ cells and granulosa cells, we found that this was perturbed by ZEN exposure, especially during the Hippo signaling pathway.

**Conclusions:** This study showed that ZEN affected the status of germ cells and granulosa cells through the Hippo signaling pathway and blocked the assembly of PFs. This research contributes to our deeper understanding of the mechanisms of toxicity in different cell types and the disruption of normal intercellular signaling by ZEN exposure.

## Introduction

Zearalenone (ZEN), also known as F-2 toxin, was first isolated and derived from moldy maize by Stob et al. in 1962 1. ZEN is a non-sterol and estrogen-active toxin produced by cereals infected with *Fusarium* 2, 3. It is reported that *Fusarium* grows best under conditions of 20-30 °C air temperature and 40% relative humidity, and that *Fusarium* produced ZEN is present in our daily environment. Since the chemical structure of ZEN is stable and can only be destroyed by exposure to 110 °C for 1 h, ZEN contamination of food has been noted in almost all countries and regions of the world 4. Therefore, ZEN is considered an important threat to both the food and animal husbandry industries, through its potential effect on the health of humans and animals.

ZEN is a known secretotoxic, genotoxic, immunotoxic, cytotoxic, hepatotoxic, and reproductive toxic chemical 5. The reproductive toxicity of ZEN severely affects fertility in most mammals 6, 7 because its chemical structure is similar to that of endogenous estrogens. The toxicity of ZEN to the female reproductive system varies with species and reproductive state (prepubertal, sexual maturity, pregnant, etc.) 8-11, or the different developmental stages of folliculogenesis (PF assembly, recruitment, follicle growth and maturation) 12-15. It has been reported that *in vitro* and *in vivo* exposure to ZEN inhibits PF assembly and also affects the quality of PFs 12, 13. In addition, Tan et al. found that the number of PFs in 4-week-old mice was significantly reduced after ZEN exposure 16. Meanwhile, studies using an *in vitro* follicle development model found that ZEN exposure significantly inhibited follicle growth and maturation 14. In addition, numerous studies have shown that ZEN exposure is extremely detrimental to folliculogenesis, but the specific mechanism by which ZEN affects early folliculogenesis remains to be explored.

The follicle is the basic functional unit of reproduction in female mammals, and PFs are the starting point for follicular development. In most mammalian species, PF number is determined before or during the neonatal period, and the entire PF pool becomes the ovarian reserve (OR) responsible for subsequent follicular growth and sexual maturation of adult female mammals 17, 18. In the mammalian embryonic ovary, primordial germ cells (PGCs) migrate from the extragonads, then proliferate through mitosis and enter meiosis, after which female germ cells become arrested during the prophase of meiosis Ⅰ 19. During development, female germ cell cysts break apart and a large number of the constituent germ cells are lost, mainly through apoptosis and autophagy 20-27. During PF assembly, the overall organization of the ovary changes, as does the cellular relationship between female germ cells and granulosa cells 28. Interaction between the two types of cells during this process is essential for maintaining the balance of perinatal ovarian function and PF assembly; signaling pathways play important regulatory roles during this transition. Studies have shown that signaling pathways such as PI3K/AKT, mTOR, and MAPK regulate important processes such as oocyte growth and development, follicle activation, and granulosa cell apoptosis 29-31. In earlier studies, the Hippo signaling pathway was found to be important for maintaining organ size 32. Recent studies have shown that the Hippo signaling pathway plays a key role in the proliferation, differentiation, and survival of granulosa cells, while the knockout of *Yap1* in granulosa cells significantly affects follicle development 33. In addition, the activation of *Yap1* plays an important role in promoting follicle growth 34. Despite advances in the study of the Hippo signaling pathway in this field, it is still unclear whether interactions between granulosa cells and germ cells are regulated during PF assembly.

Although there is evidence that ZEN exposure inhibits PF assembly, there are no studies regarding how ZEN affects the development of oocytes and granulosa cells, and the molecular mechanism that takes place between them during PF assembly. The types, states, and interactions of cells in the ovary are very different; furthermore, gene expression within different types of cells under different conditions is also very different. The majority of ovarian studies are based on whole ovaries or follicles, where the changes in gene expression of different cell types in the ovaries cannot be effectively detected. This may be because bulk RNA-seq is a method for comparing the average value of cell gene expression, and therefore the differences at the cellular level cannot be fully explored. The advent of scRNA-seq technology has enabled the detection of changes in transcriptome expression in specific types of ovarian cells, determination of a cell development map, and establishment of the network of interactions between different cells. Currently, scRNA-seq has been used to construct a developmental map of mouse ovarian germ cells during meiotic entry and PF assembly 35, 36. In addition, Wang et al. used scRNA-seq to compare single-cell transcriptional differences between non-human primates' young ovaries and aged ovaries, providing evidence for a molecular mechanism of human ovarian aging 36. This demonstrates that we can use scRNA-seq to investigate the molecular mechanism by which ZEN affects ovarian cells.

In the present study, to determine the effects of ZEN exposure on PF assembly, we performed scRNA-seq analysis of ZEN-treated ovaries of PD0 and PD3 mice. The focus was on the molecular mechanisms by which ZEN affects female germ cells and granulosa cells during PF assembly. The current results suggest that ZEN had important effects on ovarian PF assembly and provide a target for the treatment of reproductive health problems caused by ZEN. An investigation of the ways by which ZEN affects the mechanism of interaction between germ cells and granulosa cells during PF assembly is of great interest for reducing the reproductive toxicity of ZEN, prolonging the reproductive life of females, and improving the reproductive capacity of mammals.

## Results

### Identification of cell population types within mouse ovaries of ZEN treatment and normal groups

**Figure 1:**
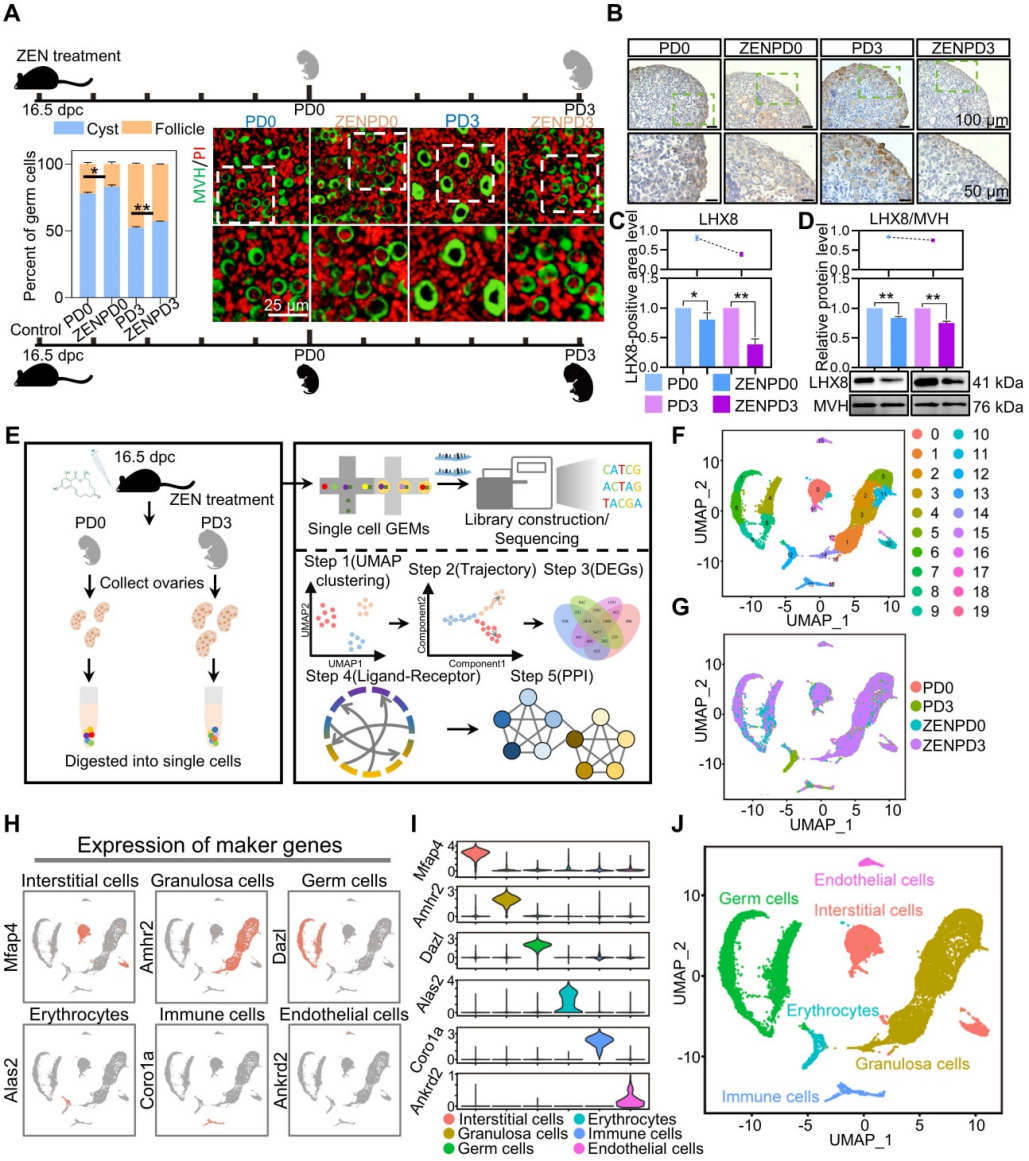
** Single-cell sequencing results and identification of cell cluster types in ovaries.** (A) After ZEN treatment, representative images of ovaries of PD0 and PD3 in the control and ZEN treatment groups. The germ cells and the nuclei were stained with MVH (green) and PI (red), respectively (right). Percentages of oocytes in cysts and PFs in the control and ZEN treatment groups in the indicated stages (left). (B) LHX8 staining of ovaries in control and treatment groups. (C) Percentage of LHX8-positive area in the ovaries of the control and treatment groups. (D) Detection of LHX8 protein expression levels by Western blot analysis. MVH was used as the loading control. (E) Schematic diagram of the scRNA-seq sample preparation process and analysis procedure. (F) UMAP map of all ovarian cells. (G) The distribution of ovarian cells in the four groups PD0, PD3, ZENPD0, and ZENPD3. (H) Expression of marker genes on UMAP map. (I) Violin chart showing the expression of specific marker genes in different cell types. (J) Cell type indicator chart. The percentage of each group is presented as the mean ± standard deviation. All experiments were repeated at least three times (*P < 0.05; **P < 0.01).

In order to study the effects of ZEN exposure on PF assembly, 40 μg/kg ZEN was administered daily to pregnant mice from 16.5 days post coitum (dpc), and the ovarian development of PD0 and PD3 offspring was examined (Figure 1A). The results of immunohistochemistry showed that ZEN exposure significantly increased the ratios of germ cells in cysts at PD0 and PD3 when compared with that of control groups (Figure 1A). This indicated that germ cell cyst breakdown was inhibited after ZEN exposure. Moreover, both the RNA and protein levels of* Lhx8* (a key transcriptional factor of PF assembly) were significantly decreased compared with that of the control groups (Figure 1B-D).

Results also showed that ZEN exposure affected the dynamic gene expression of ovarian cells during PF assembly. ZEN-treated PD0 and PD3 murine ovaries were prepared into single-cell suspensions and scRNA-seq was then performed (Figure 1E). Low-quality cells were filtered based on the number of genes per cell and the count of each gene. In total, 18 496 ovarian cells were obtained, which included 4 526 cells for PD0, 4 886 cells for PD3, 4 248 cells for ZENPD0, and 4 836 cells for ZENPD3, respectively; the median number of genes per cell ranged from 2 918 to 4 142 (Figure S1A-B). Subsequently, we integrated four samples and generated 20 cell clusters through Uniform Manifold Approximation and Projection for Dimension Reduction (UMAP) and Principal Component Analysis (PCA; Figure 1Fand Figure S2A). Following UMAP analysis, there was no obvious batch effect among the four samples (Figure 1G). Then, according to heatmap results, we divided the entire cell population into six cell types (Figure S2B). In order to better discriminate and classify ovarian cell populations, we used bubble charts to show the expression of more specific marker genes in different cell clusters 36 (Figure S2C). The germ cell cluster was confirmed by *Ddx4* and *Dazl* marker genes; the granulosa cell cluster by *Kitl* and *Amhr2*; interstitial cell cluster by *Mfap4* and *Nr2f2*; endothelial cell cluster by *Egfl7* and *Aplnr*; erythrocytes cluster* by Alas2* and *Rhd*; and the immune cell cluster by *Elane* and *Mpo* 36 (Figure S2D). Using marker gene expression in each cell cluster (Figure 1H-I), we divided the ovarian cells into six cell types (Figure 1J).

### High-resolution analysis of the effect of ZEN exposure on female germ cells

**Figure 2:**
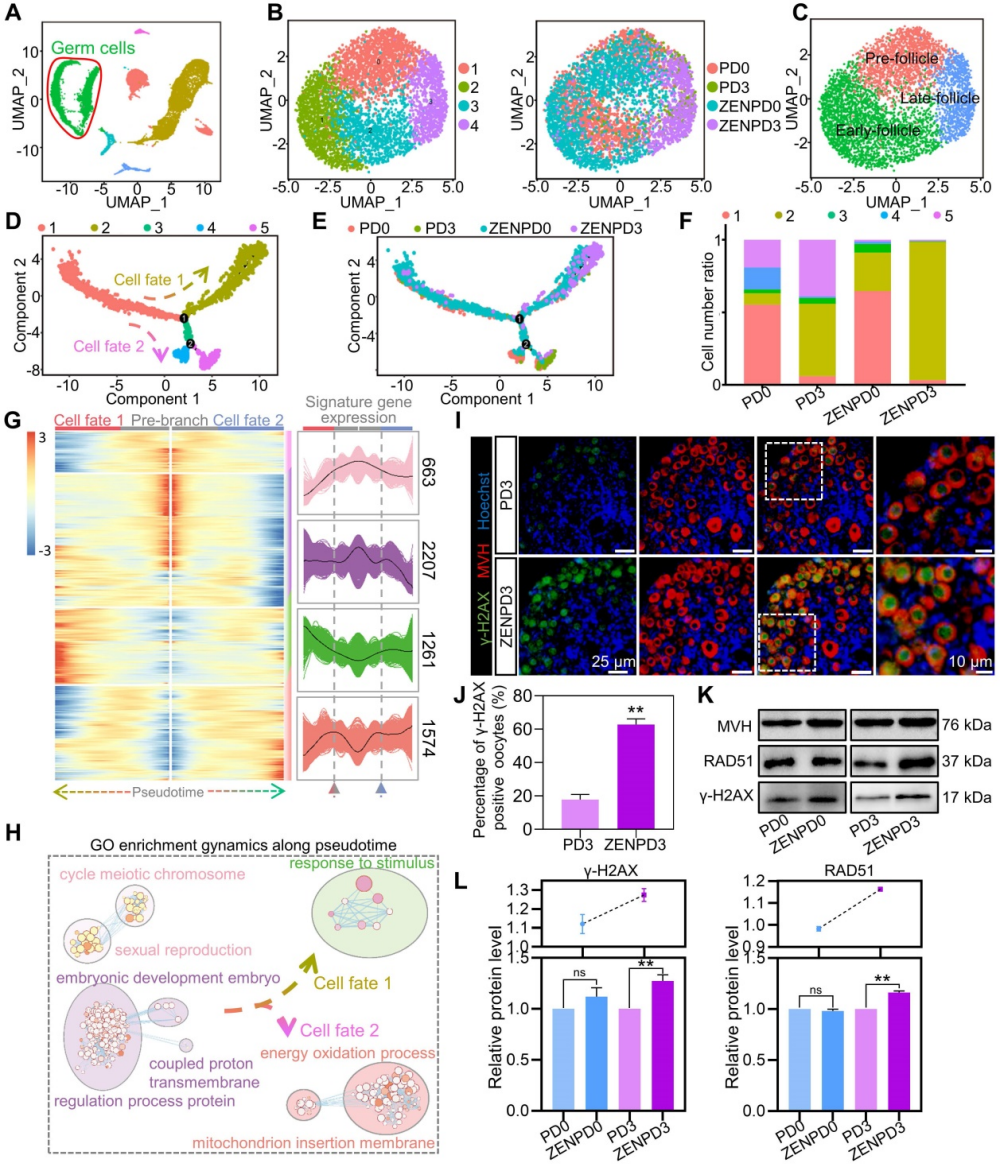
** Analysis of the heterogeneity of germ cell subsets.** (A) Extraction of germ cell clusters in the UMAP map from all ovarian cells. (B) Subpopulation of germ cell clusters (left), UMAP diagram of the germ cell subpopulations of PD0, PD3, ZENPD0, and ZENPD3 (right). (C) Germ cell clusters are divided into Pre-, Early-, and Late-follicle, and marked with different colors. (D) Single-cell pseudotime developmental trajectory of germ cells, which are colored according to cell development state. (E) The developmental trajectories of different samples of PD0, PD3, ZENPD0, and ZENPD3 in pseudotime. The cell color represents four groups of different samples. (F) Proportion of the five cell states of the germ cells at PD0, PD3, ZENPD0, and ZENPD3. The color of the histogram is determined by the cell state of the pseudotime analysis result. (G) The heatmap shows the gene expression changes of germ cells in the two cell fate branches at point 1 (left). The graph shows the expression of the genome in the pseudotime trajectory (right). (H) GO term dynamics along pseudotime. (I) MVH (red) and γ-H2AX (green) were used for IF in PD3 ovaries. (J) The percentages of γ-H2AX positive cells to the total oocytes in the control group and ZEN treatment group. (K) Representative Western blots of MVH, RAD51, and γ-H2AX. (L) Protein quantitative analysis of γ-H2AX and RAD51. The percentage of each group is presented as the mean ± standard deviation. All experiments were repeated at least three times (*P < 0.05; **P < 0.01).

In order to analyze cell heterogeneity of female germ cells at different stages after ZEN exposure, we extracted germ cell clusters for subpopulation analysis (Figure 2A). Cell cycle effects were eliminated from the germ cell clusters (Figure S3A), then UMAP projection was performed with germ cell clusters from the four groups (Figure 2B). We identified those genes specifically expressed in the four cell clusters (Figure S3B). Next, we displayed a series of typical germ cell specific marker genes in a feature plot in order to assign the four different cell clusters to different stages of PF assembly: *Sycp3* and *Ppia* were labeled Pre_follicle; *Lhx8* and *Eif4a1* were marked Early_follicle; *Gdf9* and *Ooep* were marked Late_follicle 36 (Figure S3C). We found that Pre_follicle cells were mainly distributed in cluster 0, Early_follicle mainly in clusters 1 and 2, and Late_follicle mainly in cluster 3 (Figure 2C). We then combined the Gene Ontology (GO) function analysis and clustering results of specifically expressed genes in different clusters, which also emphasized the reliability of our clustering (Figure S3D-E).

In order to clarify the effects of ZEN exposure on germ cells during PF assembly, the pseudotime series of all female germ cells was investigated 37. We observed that there were two branches in the whole female germ cell lineage trajectory, of which branch 1 occupied an important position in the whole trajectory, which determined two different cell fates (Figure 2D). Next, we used *Lhx8*, *Eif4a1*, *Gdf9*, and *Ooep* to clarify the development map of germ cells on the pseudotime trajectory (Figure S3F). Results showed that most of State1 belonged to female germ cells in the prophase of PF assembly. Importantly, both the pseudotime development trajectory and the stacking diagram showed that ZEN exposure increased the number of female germ cells that developed toward State2 at PD0 and PD3 (Figure 2E-F). According to the pseudotime development trajectory, we compared the changes of gene expression at the three branches (pre-branch: State1; cell fate 1: State2; cell fate 2: State3), and obtained four different gene sets (Cluster1: 663; Cluster2: 2207; Cluster3: 1261; Cluster4: 1574), the expression of which changed significantly along with cell trajectory (Figure 2G). We then used GO enrichment to study the gene function of these differentially expressed genes (DEGs). Before branch1, we found that the gene set was enriched in the GO terms related to cell cycle. For cell fate 1, we observed an enriched GO term related to cell stimulation of DNA damage (Figure 2H and Figure S3G). In cell fate 2, we observed that the gene set was enriched in GO terms related to energy oxidation 38 (Figure 2H and Figure S3G). These results indicated that DNA damage may be a key factor by which ZEN inhibits PF assembly. To better reveal the roles played by significantly different genes, we observed “Oxidative phosphorylation; Cellular senescence; Cell cycle; Focal adhesion, and Hippo signaling pathway, etc.” via the Kyoto Encyclopedia of Genes and Genomes (KEGG) enrichment analysis (Figure S4A). Furthermore, we analyzed branch 2 and found that the significantly different genes of the two States were mostly related to the meiotic cell cycle, which is consistent with the fate map of PF created by Wang et al. 36 (Figure S4B-D). We suggest that ZEN exposure affected the cell fate of normal germ cells, making it impossible for them to accurately complete the process of PF assembly.

After obtaining the gene sets which demonstrated that ZEN exposure significantly affected PF assembly at different stages, we further summarized the gene regulation relationship between different germ cell clusters using SCENIC and inferred the basic regulatory mechanism of germ cell state transition. We first extracted the germ cell expression matrix from Monocle as the input matrix of SCENIC. After that, the AUCell algorithm was used to score the activity of each regulon in each cell according to the standard SCENIC pipeline, and a binary activity regulon matrix was obtained. As shown in Figure S4E, the cells of State2 could be clearly distinguished from the clustering of other States, which was consistent with the results of the Monocle pseudotime trajectory. In addition, we found that XRCC4 (X-ray repair cross-complementing protein 4) existed in State2 in the binary regulon matrix, which supported the GO biological process of DNA damage and repair found in the Monocle results 39. Other transcriptional factors are closely related to regulating cell growth. Finally, the double staining results of ovarian sections of MVH and γ-H2AX showed that the proportion of PD3 γ-H2AX positive oocytes in the ZEN treatment group was significantly higher than that in the PD3 control group (Figure 2I-J). According to Western blot analysis, ZEN exposure significantly increased the protein levels of γ-H2AX and RAD51 in oocytes at PD3 (Figure 2K-L). These results further showed that ZEN exposure could cause DNA damage to oocytes during PF assembly.

### Dissecting the change in cell fates of granulosa cells affected by ZEN exposure

**Figure 3:**
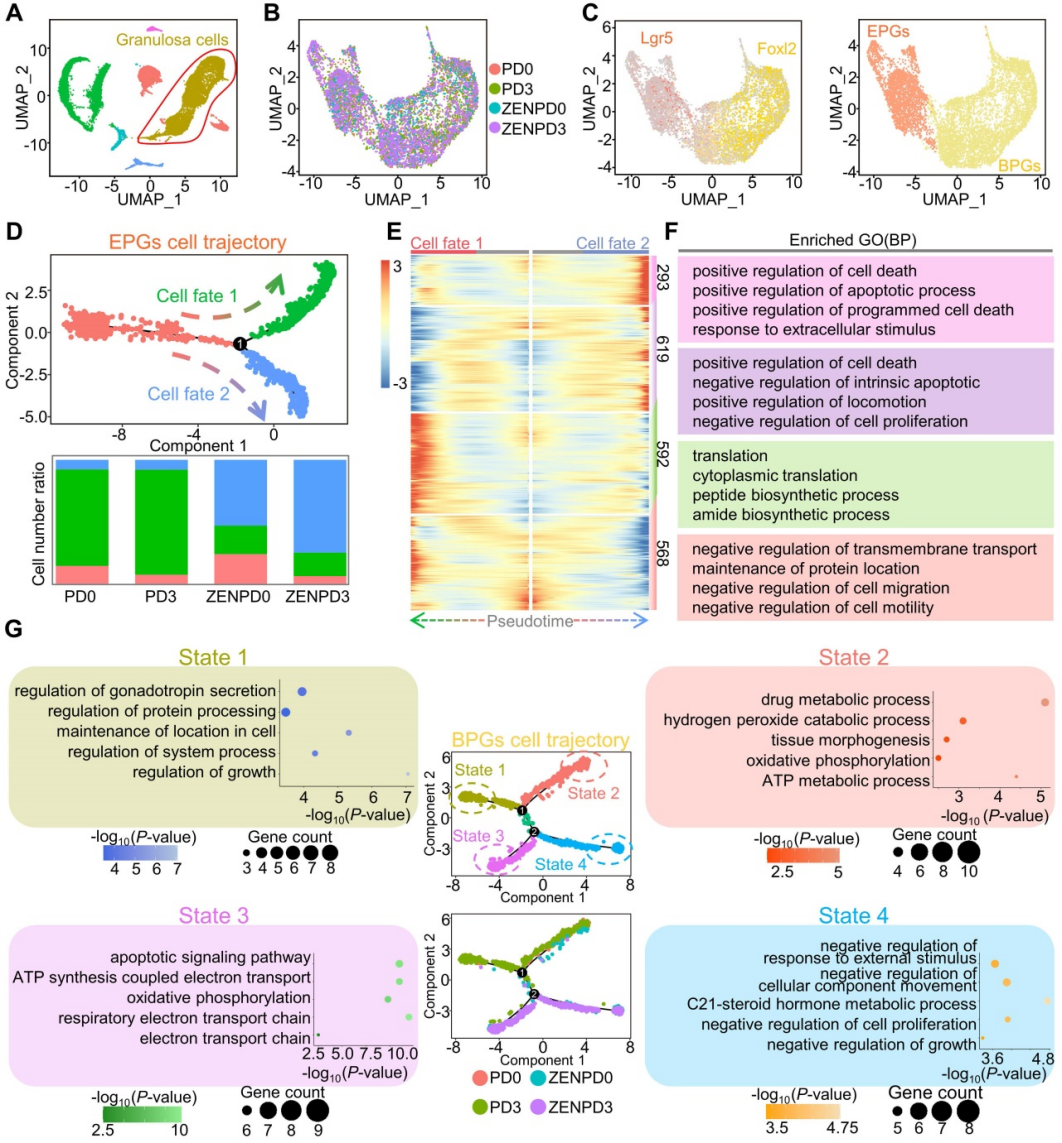
** Analysis of the heterogeneity of granulosa cell subsets.** (A) Extracted granulosa cell clusters from all ovarian cells. (B) UMAP map of PD0, PD3, ZENPD0, and ZENPD3 granulosa cell subpopulations. (C) *Lgr5* and *Foxl2* were used to label granulosa cell clusters (left); granulosa cell clusters were divided into two parts: EPGs and BPGs (right). (D) The pseudotime developmental trajectory of a single cell of EPGs (upper part). The ratio of the three cell states of the EPGs of PD0, PD3, ZENPD0, and ZENPD3 (lower part). The color depends on the state of cell development. (E) Heatmap showing the gene expression changes of all EPGs in the two cell fate branches. (F) GO term enrichment results of the four gene sets (top 50). (G) Developmental trajectories of different BPGs samples of PD0, PD3, ZENPD0, and ZENPD3 in pseudotime. Representative GO terms for stage-specific genes are shown.

After discovering the effects of ZEN exposure on germ cells, we went on to explore the effects of ZEN exposure on granulosa cells. UMAP analysis was performed on granulosa cell clusters to investigate whether ZEN exposure perturbed gene expression in granulosa cells (Figure 3A). Since the study of Niu et al. found two different pre-granulosa cell differentiation pathways, we did not perform elimination of cell cycle effects to prevent this step from affecting data accuracy (Figure S5A). After subpopulation analysis of granulosa cell clusters, we identified a total of 10 cell clusters (Figure S5B). The four groups of samples were evenly distributed in the UMAP chart (Figure 3B). According to reports, we know that *Lgr5* is a specific marker gene for epithelial pre-granulosa cells (EPGs), and *Foxl2* is a specific marker gene for bipotential pre-granulosa cells (BPGs; Figure 3C) 40-42. The heat map and violin map of different genes in each cell cluster could confirm the accuracy of our classification of granulosa cell types (EPGs: clusters 0, 6, and 8; BPGs: clusters 1, 2, 3, 4, 5, 7, and 9; Figure S5C-D). Moreover, the comparison of shared DEGs and GO terms also showed that clusters 0, 6, and 8 shared more DEGs and GO terms (Figure S5E-F). These results ensured the feasibility of our deep analysis of granulosa cells.

In order to reconstruct the pseudotime trajectory of the pre-granulosa cells during PF assembly, we performed Monocle analysis on EPGs and BPGs. After constructing cell lineage trajectories for EPGs, we observed that EPGs divided into two branches in this process (Figure 3D, upper). Before branching, the EPGs of all samples were contained, while Cell fate1 mainly contained EPGs of the control group, and Cell fate2 mainly contained EPGs after ZEN exposure (Figure 3D, below). Next, in order to gain branch-specific gene expression profiles of the pseudotime trajectory, we compared gene expression profiles in the three states and observed four gene sets (cluster1: 293; cluster2: 619; cluster3: 592; cluster4: 568; Figure 3E). In the gene set (cluster1 and cluster2) in Cell fate2, we observed enriched GO terms related to apoptosis. In the gene set (cluster3) in Cell fate1, we observed enriched GO terms related to translation. For the gene set before branching (cluster4), GO terms related to cell migration were enriched (Figure 3F). This evidence indicated that ZEN exposure might induce apoptosis in EPG cells. After pseudotime analysis of BPGs, we observed that the control group and the treatment group were distributed in different branches. We performed GO term enrichment analysis on the DEGs of the cells on the four branches and found the following: GO terms of “regulation of gonadotropin secretion” and “regulation of protein processing” were enriched on State1; GO terms of “oxidative phosphorylation; ATP metabolic process, etc.” were enriched on State2; GO terms “apoptotic signaling pathway, etc.” were enriched on State3; and the GO terms “negative regulation of response to external stimulus, etc.” were enriched on State4 (Figure 3G). These results suggested that ZEN exposure induced apoptosis in granulosa cells. In addition, we performed KEGG enrichment analysis of the significantly different genes in EPGs and BPGs, and enriched them into signal pathways related to cell growth such as Focal adhesion, Cell cycle, and the Hippo signaling pathway (Figure S5G). In order to demonstrate the reliability of our data, we performed TUNEL staining on the PD3 ovarian sections of the control groups and the treatment groups, respectively (Figure S6A). After statistical analysis, it was found that the number of TUNEL-positive granulosa cells in PD3 ovaries exposed to ZEN was significantly higher than that in the control groups (Figure S6B). In addition, we stained for Caspase3, as shown in Figure S6C, which showed that there was a higher percentage of cells expressing apoptotic genes in ZEN-exposed PD3 ovaries. Western blot analysis results also demonstrated that ZEN exposure could increase the expression levels of apoptotic BAX/BCL2 and Caspase3 in granulosa cells (Figure S6D). In summary, ZEN exposure promoted granulosa cell apoptosis and inhibited PF assembly.

### ZEN exposure affected intercellular communication between female germ cells and granulosa cells

**Figure 4:**
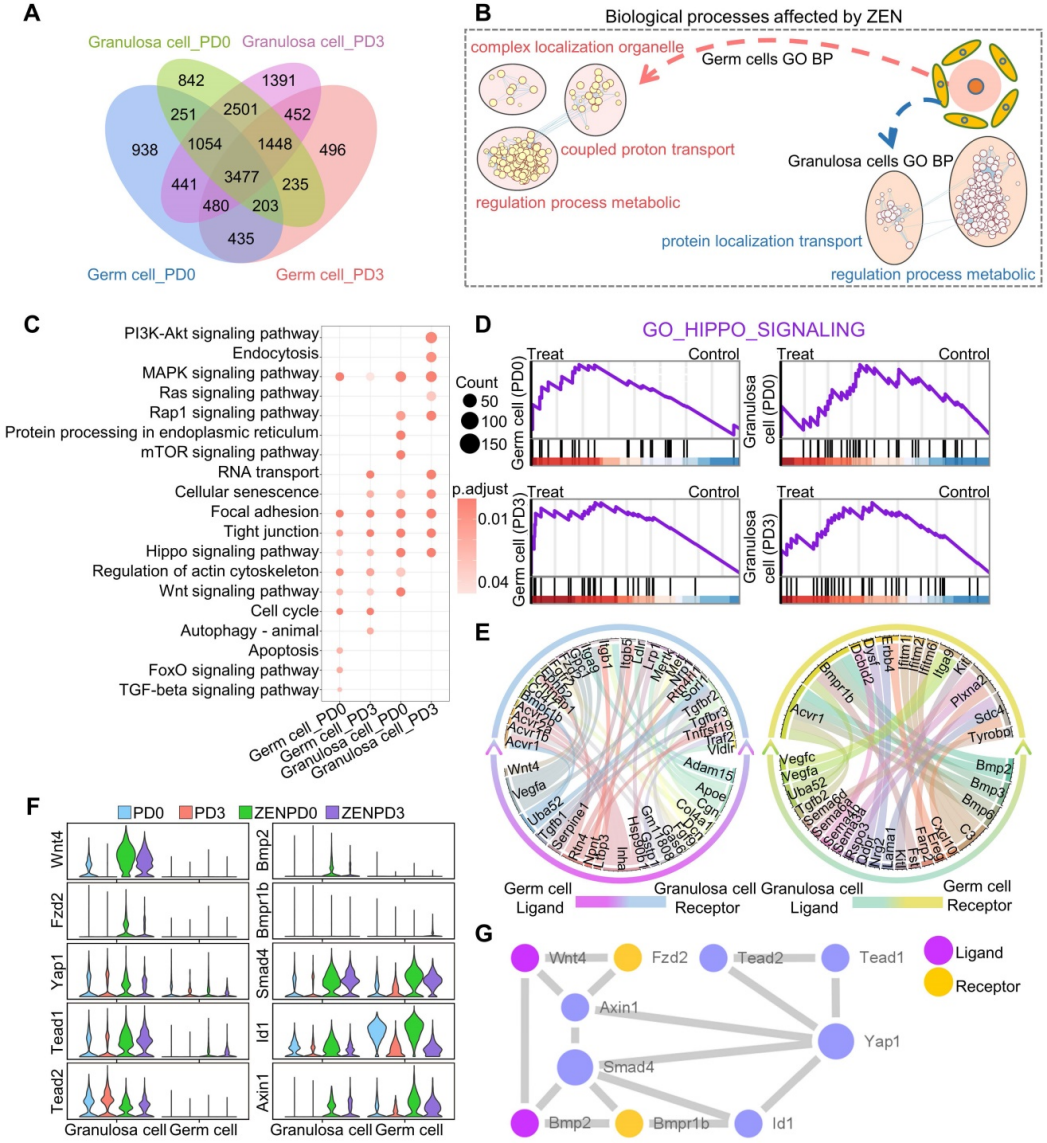
** Analysis of differential gene sets of germ cells and granulosa cells at different stages.** (A) Venn diagram showing the different genes between germ cells and granulosa cells at different stages. (B) GO_BP enrichment results of different genes of granulosa cells and germ cells at different stages. (C) Bubble chart shows the KEGG enrichment results of different genes in granulosa cells and germ cells at different stages. (D) GSEA of the Hippo pathway. (E) Signal transduction between germ cell ligands and granulosa cell receptors (left). Signal transduction between granulosa cell ligands and germ cell receptors (right). (F) Violin chart showing the expression of key genes in different cell populations. (G) Protein interaction network diagram of Hippo signaling.

Since the interaction of germ cells and granulosa cells plays an important role in PF assembly, we next investigated how ZEN exposure affected the communication between germ cells and granulosa cells. After identification of the DEGs between germ cells and granulosa cells during different periods, we noted that most DEGs were common between the four sets (Figure 4A). This showed that ZEN exposure affected germ cells and granulosa cells at different stages by affecting many similar genes. To further understand the transcriptome differences between different cells, we conducted GO analysis. The results showed that DEGs between granulosa cells and germ cells were all enriched in the “regulation process metabolic” GO term (Figure 4B). After examining further GO terms, we found that granulosa cell DEGs affected by ZEN exposure were enriched in GO terms related to cell cycle and apoptosis (Figure S7A). Furthermore, the DEGs of germ cells were enriched in GO terms related to DNA damage, and cell development and growth (Figure S7B). Through KEGG enrichment of gene sets of different groups, we found that “MAPK, Focal adhesion, tight junction and Hippo signaling pathway” were significantly enriched in the four groups (Figure 4C). In addition, the results of Gene Set Enrichment Analysis (GSEA) indicated that the gene sets affected by ZEN were generally up-regulated in the Hippo signaling pathway (Figure 4D).

Next, in order to further explore the interactive relationship between granulosa cells and germ cells, we used CellPhoneDB to define the ligand-receptor relationship between them, and displayed this using a chord diagram (Figure 4E) 43. Combined with the results of the difference analysis, we found ten key genes in the Hippo signaling pathway, the gene expressions of which are shown to be under the influence of ZEN in Figure 4F and Table S2. It is worth noting that although *Wnt4*, *Fzd2*, *Bmp2*, and *Bmpr1b* as ligands and receptors for germ cells and granulosa cells play certain roles in the upstream section of the Hippo signaling pathway, they are also key genes in the Wnt signaling pathway and the TGF-beta signaling pathway. With regard to the Wnt and TGF-beta signaling pathways, GSEA results revealed that most of the genes in the gene sets were up-regulated in the two cell types affected by ZEN exposure at different periods (Figure S7C-D). Finally, we described the relationships among the ten proteins through protein-protein interaction network analysis (Figure 4G).

**Figure 5:**
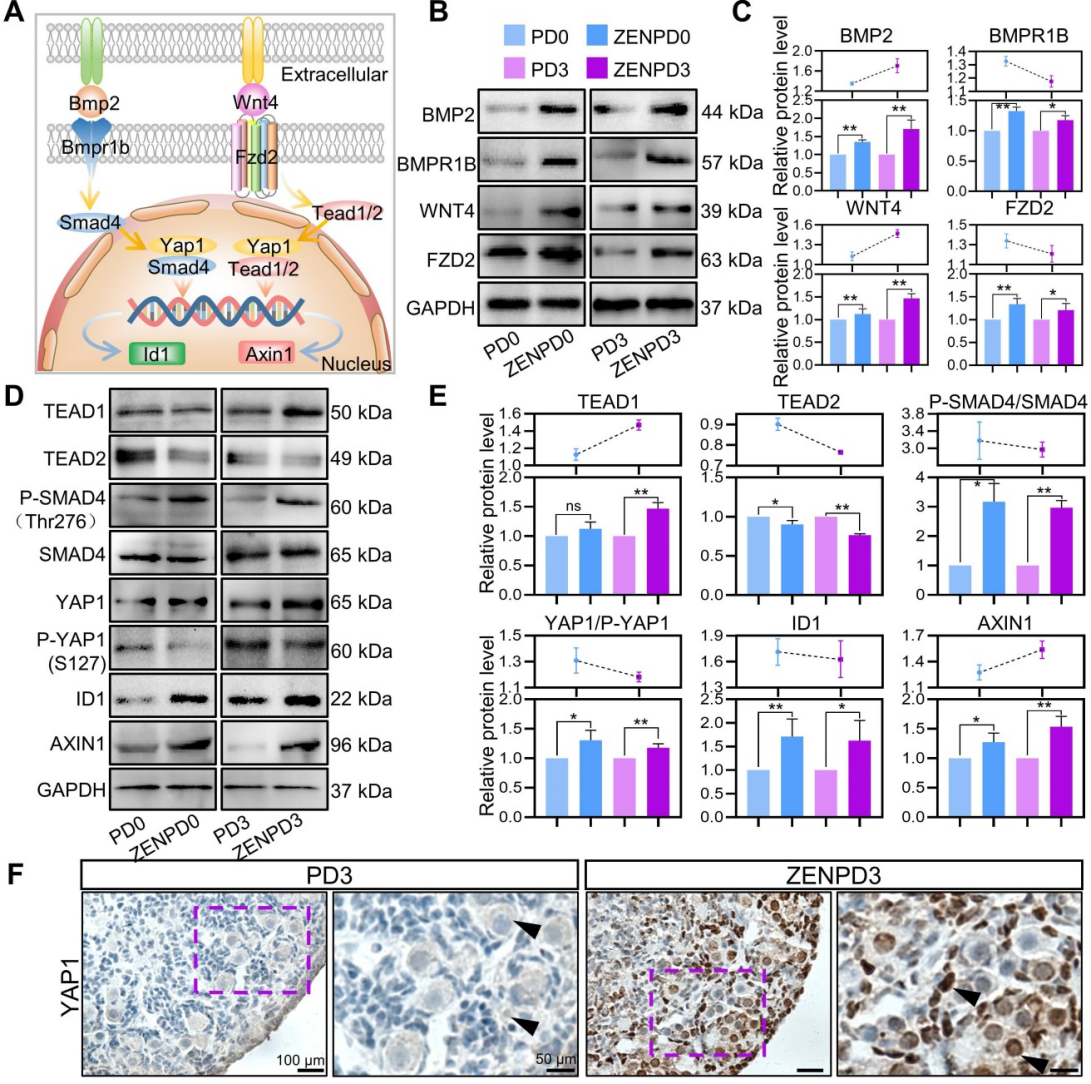
** Key Gene Expression Pattern of the Hippo Signaling Pathway.** (A) Schematic diagram showing the signal transduction pathway of germ cells and granulosa cells. (B-C) Western blot results of BMP2, BMPR1B, WNT4, FZD2, and GAPDH. (D-E) Western blot results of TEAD1, TEAD2, SMAD4, P-SMAD4, YAP1, P-YAP1, ID1, AXIN1, and GAPDH. (F) YAP1 staining of PD3 ovarian tissue in the control group and the treatment group. The percentage of each group is presented as the mean ± standard deviation. All experiments were repeated at least three times (*P < 0.05; **P < 0.01).

We used a pattern diagram to describe the position of the ten key proteins analyzed in the cells (Figure 5A). In order to further verify the accuracy of our analysis, we used Western blot analysis to detect the changes in expression of these ten key proteins in the ovaries. Results showed that the ligand-receptor expression of BMP2 and BMPR1B in granulosa cells, and WNT4 and FZD2 in female germ cells, were significantly increased by the influence of ZEN exposure in different periods (Figure 5B-C; *P* < 0.05 or *P* < 0.01). Expression of the key genes TEAD1, SMAD4, YAP1, ID1, and AXIN1 in the Hippo signaling pathway was also significantly increased under the influence of ZEN, and the expression of TEAD2 was significantly decreased (Figure 5D-E; *P* < 0.05 or *P* < 0.01). In addition, we located YAP1 using immunohistochemistry (IHC) and found that ZEN treatment caused YAP1 to enter the nucleus (Figure 5F). This showed that the results of our bioinformatics analysis were credible.

### Downregulation of *Yap1* reduced the harmful effects of ZEN exposure on the ovaries

In order to prove that the expressions of *Axin1* and *Id1* were regulated by the Hippo signaling pathway, we added Verteporfin (VP; an inhibitor of Hippo signaling) 44 to the culture medium when culturing neonatal mouse ovaries *in vitro*, and found that the expression levels of AXIN1 and ID1 were significantly reduced (Figure S8A; *P* < 0.05 or *P* < 0.01).

**Figure 6:**
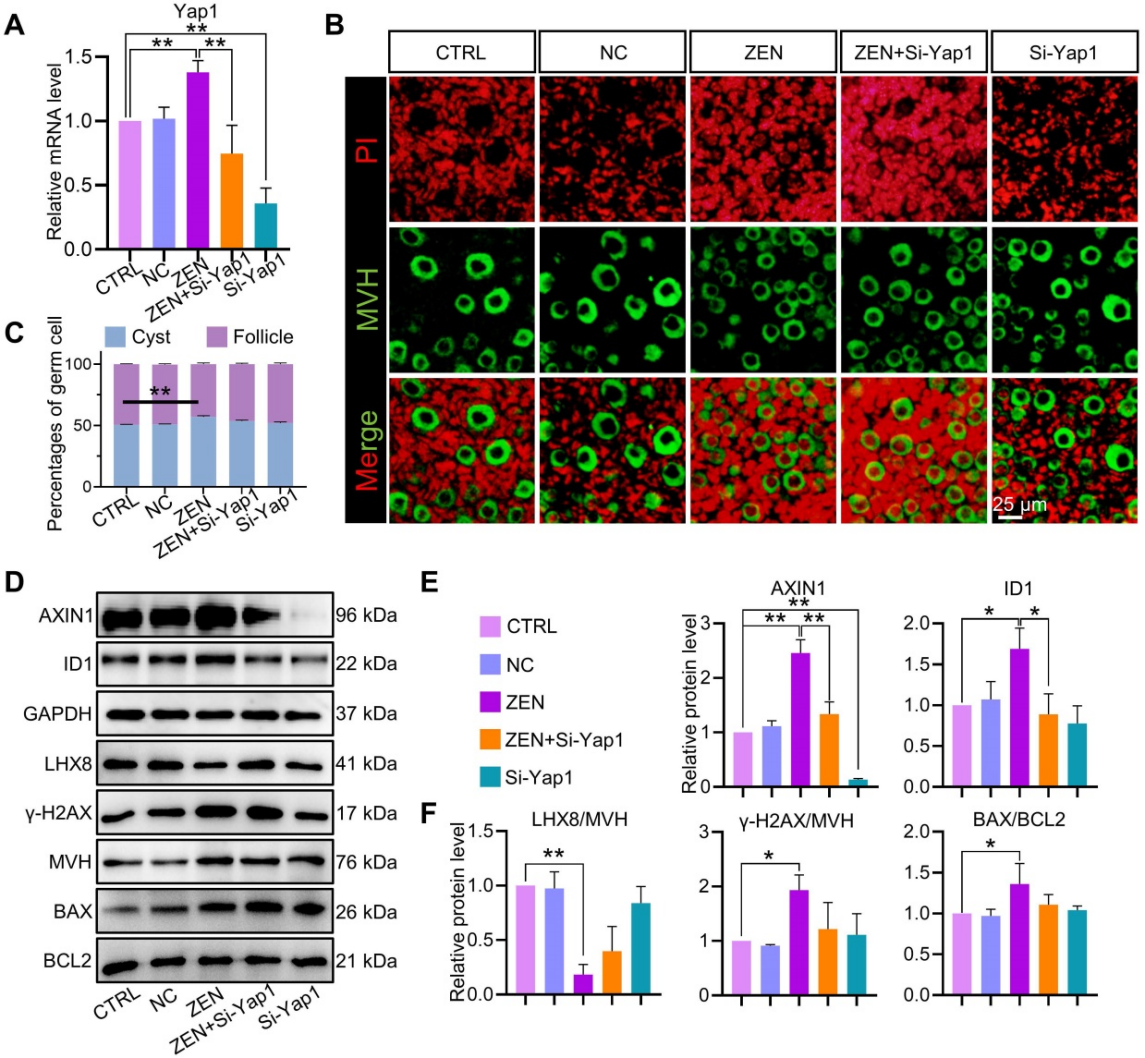
***Yap1* RNAi relieved the inhibition of ZEN on PF assembly.** (A) *Yap1* mRNA levels in the control and treatment groups. (B) Representative images of PD3 ovaries in the control, negative control, ZEN treatment, ZEN plus *Yap1* siRNA, and *Yap1* siRNA groups. Germ cells and nuclei were stained with MVH (green) and PI (red), respectively. (C) The percentage of oocytes in cysts and PF in each group at the specified stage. (D-F) Western blot and quantitative analysis results of AXIN1, ID1, GAPDH, LHX8, γ-H2AX, MVH, BAX, and BCL2. The percentage of each group is presented as the mean ± standard deviation. All experiments were repeated at least three times (*P < 0.05; **P < 0.01).

To confirm the key role of up-regulated *Yap1* in ZEN-induced ovarian toxicity, RNA interference (RNAi) of *Yap1* was performed during *in vitro* ovarian culture (Figure S8B). The results of RT-qPCR showed that RNAi did reduce the expression of *Yap1* in the ovaries (Figure 6A). In addition, we found that after *Yap1*-RNAi of the ovaries cultured *in vitro*, the inhibitory effects of ZEN on PF assembly were reduced (Figure 6B-C). Most importantly, the expression levels of AXIN1 and ID1 returned to normal levels similar to the control group (Figure 6D-E). Finally, we used Western blot analysis to detect those proteins related to PF assembly (LHX8), DNA damage (γ-H2AX), and apoptosis (BAX/BCL2), and found that knocking down the expression level of *Yap1* could up-regulate the expression of LHX8 protein and down-regulate the expression of γ-H2AX and BAX/BCL2 proteins (Figure 6D-F). These results indicated that *Yap1* played an important role through the Hippo signaling pathway during the effects of ZEN-mediated PF assembly.

## Discussion

In most female mammals, perinatal PF formation is critical for ovarian development, and the mechanisms underlying PF assembly are complex. During PF assembly, the ovaries are very fragile and vulnerable to foreign substances that can cause irreparable damage to ovarian development 45. Among these, research related to endocrine disrupting chemicals (EDCs) is emerging as a hot topic in the field of reproduction, with the following environmental estrogens being the most studied: di (2-ethylhexyl) phthalate, bisphenol A, and ZEN. In particular, ZEN, an EDC with strong estrogenic properties, cause more pronounced damage to female mammals 12; however, the mechanism of action is unclear. In this study, the transcriptome dynamics of fetal ovarian development after ZEN exposure of pregnant mice, and the differential changes in signaling communication pathways, were interpreted in detail using the scRNA-seq technique. First, we analyzed the changes of female germ cell development affected by ZEN exposure using scRNA-seq with high resolution, and dissected the process of granulosa cell fate transformation after ZEN exposure. Subsequently, we also found that ZEN exposure affected the interactions between female germ cells and granulosa cells. In addition, we inhibited the expression of YAP1 using an RNA interference technique and found that the decreased expression of YAP1 could reduce the toxic effects of ZEN on PF assembly, which further revealed the important role of the YAP1*-*mediated signaling pathway in maintaining normal folliculogenesis. These results provide a new perspective for the study of the mechanism of ZEN reproductive toxicity.

Recent studies have shown that environmental estrogens and high concentrations of estradiol exposure significantly inhibit PF assembly in PD0 mouse ovaries 12, 46, 47, or in embryonic stage (E) 17.5 mouse ovaries 48. In previous reports, only bulk RNA-seq was used to explore changes in the transcriptome level of ovarian cells during PF assembly. Results obtained in this way are usually an average value of cell gene expression 12. In the current research, with the help of scRNA-seq, we determined the effect of ZEN exposure on female germ cells and granulosa cells at the transcriptome level, and redefined the toxic effects of ZEN exposure on specific types of cells. Using this method, we found that ZEN exposure significantly inhibited the assembly of PFs, and that the growth status of female germ cells and granulosa cells was significantly changed after ZEN exposure. These results further confirmed the important toxic influence of ZEN on PF assembly and provided a theoretical basis for our subsequent in-depth study.

**Figure 7:**
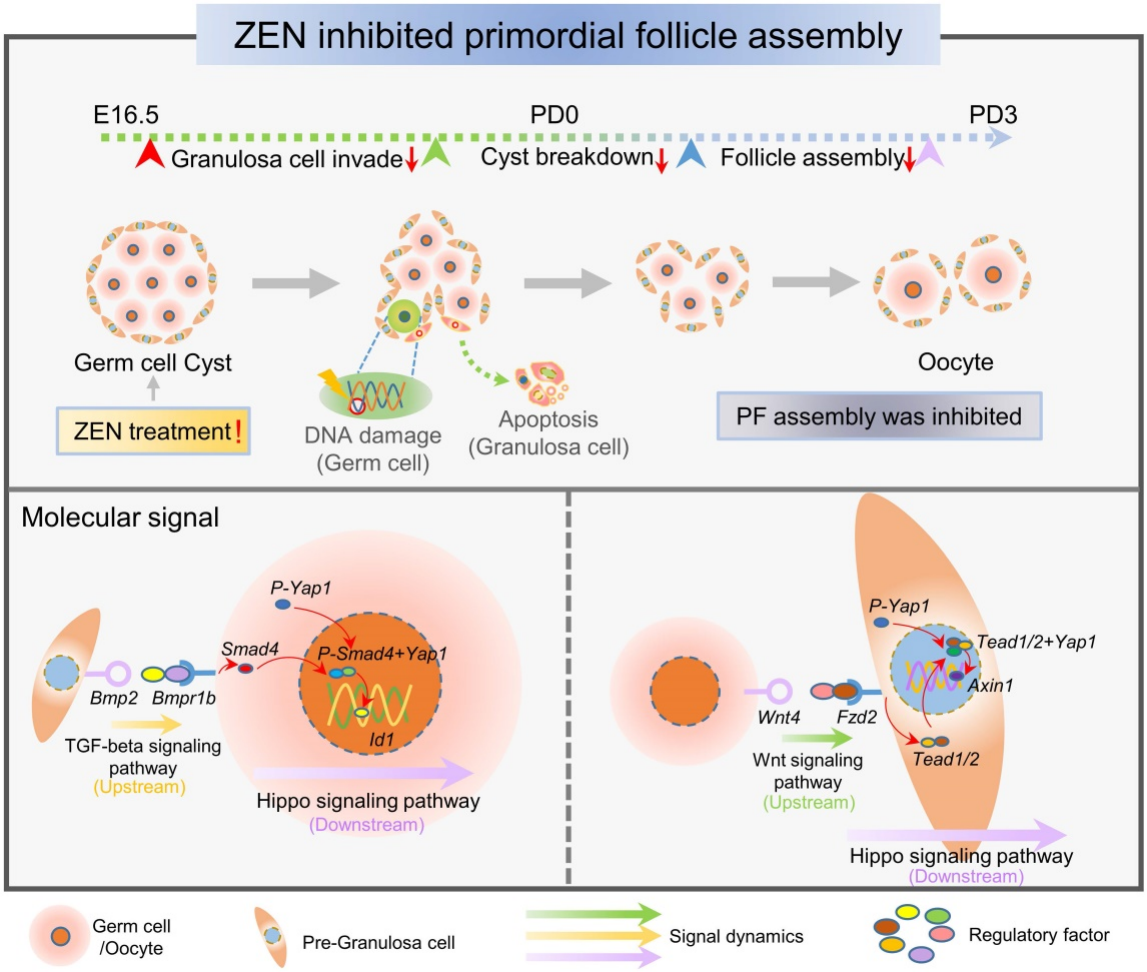
** Schematic diagram of the genetic and molecular signal dynamics that ZEN mainly affected during PF assembly.** The figure above shows that ZEN exposure led to germ cell DNA damage and granulosa cell apoptosis, thereby inhibiting the process of PF assembly. The figure below shows that the main interaction pathway between germ cells and granulosa cells was activated during this process. The main regulatory factors during the period include: *Bmpr1b*, *Bmp2*, *Wnt4*, *Fzd2*, *Yap1*, *Tead1*, *Tead2*, *Smad4*, *Axin1,* and *Id1*.

To deeply analyze the role of female germ cells and granulosa cells in this ZEN-mediated process, we used several algorithms and bioinformatics analyses. First, we used existing information to confirm the reliability of the female germ cell developmental trajectory during the perinatal period. Interestingly, we found that some ZEN-treated female germ cells were transferred to another cell fate, and their cell state was significantly different from that of normal germ cells. By analyzing different gene sets in this trajectory, we identified biological processes associated with DNA damage, which confirmed ZEN's toxicity to cause DNA damage in germ cells (Figure 7) 13. In addition, by using SCENIC analysis, we confirmed ZEN's toxicity to induce DNA damage related to transcriptional factors in germ cells. Numerous existing studies suggest that granulosa cells play an important role in PF assembly. In the present study, we identified abnormal granulosa cells from both sources after ZEN treatment, and that ZEN had a toxic effect on the state of ovarian granulosa cells during PF assembly. Based on the different gene sets of cells with different states on the granulosa cell developmental trajectory, we concluded that ZEN exposure mainly affected granulosa cell apoptosis (Figure 7). In addition, Liu et al. used RNA-seq analysis to find that ZEN exposure can affect gene expression in porcine granulosa cells, and DEGs are largely enriched in the regulatory pathways of apoptosis 49. According to reports, studies using whole-transcriptome sequencing analysis found that ZEN can induce porcine granulosa cell apoptosis through the miRNA-mRNA network 50. This is similar to our research conclusion.

During the establishment of the female ovarian reserve, the normal development of oocytes and granulosa cells, as well as normal intercellular signaling communication, are determinants of PF assembly 45. According to current research, several related signaling pathways and estrogens have been involved in the physiological processes that regulate PF assembly. Studies have shown that inhibiting the Notch signaling pathway *in vitro* can induce oocyte apoptosis, inhibit PF assembly, and affect the quality of follicles 51, 52. Wang et al. used scRNA-seq to address cellular heterogeneity during PF assembly and constructed a single-cell developmental trajectory of ovarian cells; revealing that Hippo signaling pathways may be one of the important pathways affecting PF assembly 36. Our research not only explored the *in vivo* toxicity of ZEN on PF assembly, but also identified the mechanism that ZEN affected PF assembly via the Hippo signaling pathway.

In mammalian cells, both Wnt and TGF-beta signaling pathways are regulated by the Hippo signaling pathway. In previous studies, WNT ligands and FZD receptors were shown to induce activation of the Hippo signaling pathway, thereby regulating the growth and development of cells, tissues, and organs 53, 54. In addition, it is reported that once the ligand receptor on the TGF-beta signaling pathway is activated, the SMAD complex will accumulate in the nucleus and bind to YAP, thereby activating the Hippo signaling pathway 55, 56. The important finding of the current study was that ZEN activated the Hippo signaling pathway by regulating the binding of the Wnt signaling pathway and the ligand receptor protein in the TGF-beta signaling pathway, and thus ultimately regulated PF assembly. Thereby, our study found that YAP1 expression was significantly increased in germ cells and granulosa cells after ZEN exposure and the protein started to enter the nucleus. It is suggested that ZEN affects germ cells and granulosa cells through the Hippo signaling pathway. Furthermore, in the present study, we found that* Yap1* knockdown resulted in a significant decrease in the expression of *Axin1* and *Id1*, which are essential for alleviating damage to PF assembly caused by ZEN exposure. Previous studies have shown that *Axin1* is involved in granulosa cell growth and differentiation, and *Id1* plays a key role in oocyte development 57, 58. In addition, Zhang et al. processed mouse granulosa cells with ZEN and used RNA-seq analysis to find that the Hippo signaling pathway was activated 59. Thus, the Hippo signaling pathway is an important route for ZEN regulation of PF assembly (Figure 7).

In summary, this study suggested that ZEN affected the status of germ cells and granulosa cells through the Hippo signaling pathway and blocked the assembly of PFs. This research contributes to our deeper understanding of the mechanisms of toxicity in different cell types and the disruption of normal intercellular signaling by ZEN exposure.

## Materials and methods

### Animals

The C57/BL6 mice used in this study were provided by Vital River Laboratory Animal Technology Co., Ltd (Beijing, China). All mice were housed with a daily light duration of 12 h and at a temperature of 23 ± 1 °C. Mating was arranged between 6-week-old female mice and 8-week-old male mice, and those females with a vaginal plug the next morning were considered to be 0.5 dpc. Animal breeding and experiments in this study were conducted in strict compliance with Animal Care and Use Committee of Qingdao Agricultural University.

### ZEN treatments

ZEN (Sigma, Z2125, MO, USA) was dissolved in DMSO at 40 mg/ml and stored at -20 °C in the dark. Before oral administration, ZEN was dissolved in phosphate buffer saline (PBS) at 40 μg/mL concentration. Pregnant mice of 16.5 dpc were continuously administered an oral treatment for 3 or 6 days according to body weight. The ZEN concentration was determined based on published articles 16, 59.

### Single cell library preparation and sequencing

Ovaries were collected from separate mice at PD0 and PD3. In order to obtain a sufficient number of cells and satisfy certain repeatability tests, 10 ovaries were collected from each group. Subsequently, the ovaries were minced and digested with 0.25% trypsin (Sangon Biotech, Shanghai, China, A003702) and collagenase (2 mg/ml, Sigma, C5138) for 6-8 min at 37 ℃. The solution was filtered through 40 μm cell filters (BD Falcon, USA, 352340) and washed three times with 0.04% bovine serum albumin (BSA, Sigma, A1933) in PBS to obtain a cell suspension. Cells were then stained with Trypan blue and a Countess Automated Cell Counter (Thermo Fisher Scientific, Waltham, USA) was used to detect cell concentration and viability. The average cell survival rate met the required standards.

Single-cell libraries were then prepared using a Chromium Chip B Single Cell Kit (10× Genomics, Pleasanton, CA, USA; 1000073), and Chromium Single Cell 3′ Library & Gel Bead Kit v3 (10× Genomics; 1000075) according to the manufacturer's instructions, and an Illumina NovaSeq 6000 sequencer (Illumina, San Diego, CA, USA) was used for sequencing reads with paired ends of 150 bp (PE150).

### Preliminary analysis and clustering analysis of single cell samples

We used CellRanger v.3.1.0 software to analyze the raw data and to obtain a barcode table and gene expression matrix (Table S3). At the same time, we obtained basic sequencing information through the website, such as the number of cells, the median of detected genes, and total genes detected, etc. (Figure S1A). Then, we used R (v.3.6.3) and Seurat v.3.1.5 R packages to control the quality of the data. According to the median of the genes in the sample and the percentage of mitochondrial genes, referring to the filtering criteria of the previously studied human cells, a total of 17 804 cells were obtained (Figure S1B). We then detected and visualized the relationship between the percentage of mitochondrial genes and mRNA reads, and the relationship between the number of mRNAs and mRNA reads (Figure S1C). Next, in order to avoid the influence of batch processing effects on downstream analysis, we integrated the four sets of data through “FindIntegrationAnchors” and “IntegrateData”. Subsequently, we used the “RunUMAP” function to visualize the integrated data to obtain cell clusters. In order to mark cell clusters, we used the “FindAllMarkers” function to find specific marker genes.

### Removal of dual cell effects

Potentially, two or more cells could have been in one droplet that captured the cells, which could cause one type of cell in the analysis data to show two different cell marker genes and thus affect the data quality. Since there is no corresponding algorithm for deleting double cells in Cellranger and Seurat, the R package DoubletFinder (v.2.0.3) was used to remove the effect of double cells on single-cell transcriptome data 60. First, we generated artificial doublets from existing scRNA-seq data. After that, artificial doublets were mixed with real cells. Next, we performed PCA and used the PC distance matrix to find the proportion of artificial k-nearest neighbors (pANN) for each unit. Finally, according to the expected number of doublets arrangement order and threshold pANN value, the number of double cells in PD0 was 325; the number of double cells in PD3 was 360; the number of double cells in ZENPD0 was 297; the number of double cells in ZENPD3 was 354.

### Cell cycle analysis

The heterogeneity of cell cycle phases, especially the transition between mitotic cells in S phase and G2/M phase, drives a large number of transcriptome mutations, thereby masking biological signals. This step aimed to reduce the influence of transcriptional heterogeneity of mitotic cells at different stages without affecting the overall difference between mitotic cells and non-mitotic cells that may have biological significance. We used the “CellCycleScoring” function in Seurat v.3.1.5 R package to detect the germ cell population and granulosa cell population. When the period scores of G1/S and G2/M were both < 2, we considered these cells to be aperiodic; otherwise, we considered that the cell was proliferating. After cell cycle analysis, the deviation caused by cell cycle genes was observed in granulosa cells, but the cell cycle effects of germ cells were eliminated (Figure S3A and Figure S5A).

### Single-cell pseudotime trajectory analysis

First, we used the Seurat “subset” function to pick out the germ cell cluster and the granulosa cell cluster to import into Monocle v.2.8.0, and the variable genes identified by the Seurat “FindVariableFeatures” function were used to order genes for pseudotime. Next, we used the “orderCells” function to perform dimensionality reduction and trajectory analysis on the data. For the nodes in the results of the pseudotime analysis, the branch-specific gene expression changes were calculated using the “BEAM” function. Finally, the “plot_genes_branched_heatmap” function was used to visualize the branch-heatmap of the branch-specific genes.

### Identification of differentially expressed genes

In order to identify genes that were differentially expressed in germ cells and granulosa cells after exposure to ZEN, differential expression analysis was performed using the DEsingle software package 61. The DEsingle package has a reasonable solution to the large number of zeros in scRNA-seq and the technical dropout. We used a DEsingle-corrected *P*-value to determine DEGs, and considered genes with *P*-value ≤ 0.05 to belong to DEGs.

### Analysis of the regulation of single-cell cluster transcription factors

To reveal the gene regulatory network of germ cells treated with ZEN at different stages, we used SCENIC to infer the gene regulatory network based on co-expression and motif analysis, and then analyzed the network activity in each cell to identify the cell state 62. In the current study, Monocle's single-cell RNA-seq expression matrix was used as the SCENIC input matrix. First, we used GENIE3 to infer a co-expression matrix containing potential regulatory factors. Then we used RcisTarget for motif analysis. Finally, we used AUCell to identify cells with active gene sets in the scRNA-seq data.

### GO, KEGG enrich analysis, GSEA, and protein-protein network enrichment analyses

Differentially expressed genes were selected based on the *P*-value, and we used Metascape for GO enrichment analysis. With the help of g:Profiler, Cytoscape and EnrichmentMap, the GO biological process network map was established 38, 63, 64. In addition, we used the R Bioconductor/clusterProfiler package to perform GO, KEGG, and GSEA enrichment analyses on a gene set of more than 3000 genes 65. The STRING database was used to infer protein-protein networks from different genomes.

### Ovarian culture* in vitro*

Previously, we have established a fairly mature model of ovarian development *in vitro*. The results of our *in vitro* and *in vivo* experiments are highly consistent, proving the reliability of the results of *in vitro* experiments. Combining *in vivo* and *in vitro* experiments will make the results more convincing. On the other hand, compared with traditional gene knockout methods, RNAi technology has the advantages of fast, high efficiency, strong specificity, and easy operation. In addition, inhibitor treatment has been widely used in the research of signaling pathways. In order to verify that ZEN affects the status of germ cells and granulosa cells through the Hippo signaling pathway, and affects the assembly of primitive follicles, we used RNAi and inhibitors to treat PD0 ovaries *in vitro*.

Ovaries from newborn mice were isolated and cultured in 400 µL of ovarian culture medium composed of Dulbecco's modified Eagle's medium/F12 (DMEM/F12; Hyclone, Beijing, China, SH30023.01B) and α-minimal essential medium (α-MEM; Hyclone, SH30265.01B) 1:1 supplemented with 0.23 mmol/L sodium pyruvate (Hyclone, SH40003-12), 10% fetal bovine serum (FBS; Gibco, Grand Island, NY, 10099-141), 100 IU/mL of penicillin G, and 100 mg/mL of streptomycin sulfate. Before transfection, we placed the ovaries in medium at 37 °C for 30 min to equilibrate. In addition, 1 μL of Lipofectamine™ 3000 Transfection Reagent (Lipo 3000, Thermo Fisher Scientific, L3000008) and 1 μL of 20 μmol/L *Yap1* siRNA (GenePharma, Shanghai, China; Table S4) were mixed with 50 μL culture medium, respectively, at room temperature (RT) for 5 min. We then mixed siRNA with Lipo 3000 at RT for 20 min. Finally, the mixture was added to the medium. After 6-8 h, the medium was changed to a fresh batch, and the ovaries were recovered after three days of culture. In the current study, 10 μM Verteporfin (VP, MedChemExpress, Shanghai, China, CL318952) and 30 μM ZEN were used to treat the ovaries, and half of the medium was changed every other day 44.

### Western blotting

The collected ovaries were treated with RIPA lysate solution for protein extraction (Beyotime, Nantong, China, P00113B). Then the samples were mixed with SDS-polyacrylamide gel electrophoresis (SDS-PAGE) loading buffer and placed in boiling water for 5 min to denature the protein. Next, the sample proteins were separated using SDS-PAGE gel electrophoresis and transferred to a PVDF membrane (Millpore, Darmstadt, Germany, ISEQ00010). After blocking with 5% BSA for 2 h at RT, the PVDF membrane and primary antibody (Table S1) were incubated for 7 h at 4 °C. Next, the membrane was washed with TBST, followed by incubation with the secondary antibody for 2 h at RT (Table S1). Finally, we used the BeyoECL plus kit (Beyotime, P0018) to detect the protein signal, and used AlphaView SA software (ProteinSimple, San Jose, California, USA) to analyze the data. The protein of each sample was obtained from 8 ovaries at a time *in vivo* / *in vitro* and repeated at least 3 times.

### RNA extraction and qRT-PCR

In this experiment, we used the EASYspin Plus RNAprep pure Micro Kit (Aidlab, Beijing, China, RN28) to obtain total RNA from the ovaries. Afterwards, reverse transcription was performed using an RNA reverse transcription kit (TransGen, Beijing, China, AT311-03) according to the instructions. The reaction conditions were 25 °C for 10 min, 42 °C for 15 min, and 85 °C for 30 s. The cDNA was stored at -20 °C. RT-qPCR was performed using SYBR^®^ Premix Ex Taq™ II (Takara, Dalian, China, RR820A) on a Roche 480 light cycler real-time PCR instrument (Roche, Germany). Each sample procedure was performed strictly in accordance with the standard, and at least three independent replicate samples were required to calculate the data. Relative quantitative PCR data analysis was performed using the difference multiple = 2^-∆∆ct^ method. The RNA of each sample was obtained from 6 ovaries every time* in vitro* and repeated at least three times.

### Immunostaining of ovaries

For immunostaining, we fixed the collected intact ovaries in 4% paraformaldehyde (PFA) at 4 °C overnight. They were then dehydrated and embedded in paraffin according to standard histological embedding procedures. Afterwards, 5 μm thick slices were cut from each ovary. Before use, the slides were deparaffinized with xylene, hydrated with different concentrations of ethanol, and then placed in sodium citrate at 96 °C for 10 min. After slides were cooled to RT and blocked with BDT (3% BSA, 10% normal goat serum in TBS) for 30 min, they were incubated with the primary antibody (Table S1) at 4 °C overnight. Slides were then incubated with the secondary antibody for 30 min (Table S1). Finally, Hoechst 33342 (Beyotime, C1022) or PI (Solarbio, P8080, Beijing, China) was used as a nuclear stain. Oocytes in cysts and follicles were distinguished based on existing research, and were counted from every five ovarian serial sections 66, 67.

For IHC, we used the same procedure as above before using 3% H_2_O_2_ for 10 min. We then used BDT to block the reaction for 45 min. Subsequently, slides were incubated with the primary antibody overnight at 4 °C; with the secondary antibody for 1 h at RT (Table S1); and finally stained with peroxidase substrate and counterstained with hematoxylin. In the immunostaining experiment, the slices were taken from one ovary at a time *in vivo*/*in vitro*, repeated at least three times.

### TUNEL staining

In this study, a TUNEL BrightGreen Apoptosis Detection Kit (Vazyme, Nanjing, China, A112) was used for TUNEL staining experiments 68. After secondary antibody incubation was completed according to the immunofluorescence protocol, proteinase K was used for incubation at RT for 15 min. We then used Equilibration Buffer (5× Equilibration Buffer and deionized water; 1:5 dilution) to process the sample at RT for 30 min. Subsequently, a TUNEL reaction mixture (Recombinant TdT Enzyme: BrightRed Labeling Mix: 5× Equilibration Buffer: ddH_2_O; 1:5:10:34 dilution) was added. Finally, we used Hoechst 33342 as a nuclear stain.

### Statistical analysis

Each data set was obtained from at least three independent replicates. The data are shown as mean ± SD. GraphPad Prism 8.0 software (GraphPad Software, San Diego, CA) was used to determine the statistical significance of the data. Data analysis was performed using the *t*-test or one-way analysis of variance (ANOVA). Significant and highly significant differences are shown as * *P* < 0.05 and ** *P* < 0.01.

## Supplementary Material

Supplementary figures and tables.

## Abbreviations

ANOVA: one-way analysis of variance
BPGs: bipotential pre-granulosa cells
dpc: days post coitum
DEGs: differentially expressed genes
E: embryonic stage
EPGs: epithelial pre-granulosa cells
GO: Gene Ontology
KEGG: Kyoto Encyclopedia of Genes and Genomes
OR: ovarian reserve
PCA: Principal Component Analysis
PD3: postnatal day 3
PE150: paired ends of 150 bp
PGCs: primordial germ cells
PF: Primordial follicle
scRNA-seq: single-cell RNA sequencing
UMAP: Projection for Dimension Reduction
ZEN: Zearalenone
