# Supplementary material for: Single-cell transcriptomic profiling provides insights into the toxic effects of Zearalenone exposure on primordial follicle assembly
Source: Theranostics. 2021 Mar 5;11(11):5197–213. doi: 10.7150/thno.58433 (PMC8039963; doi:10.7150/thno.58433)
Supplement: Supplementary file 1 — Supplementary figures and tables. [file thnov11p5197s1.pdf]

Supplemental Figures

A

Sample info	PD0	ZENPD0	PD3	ZENPD3
Estimated Number of Cells	5000	5000	5000	5000
Valid Barcodes	97.4%	97.5%	97.5%	98.0%
Mean Reads per Cell	121,362	119,385	112,621	108,866
Median Genes per Cell	3,134	3,997	2,918	4,142
Total Genes Detected	22,280	22,799	21,795	22,677
Reads Mapped to Genome	92.4%	95.5%	93.4%	95.1%
Reads Mapped Confidently to Transcriptome	66.6%	64.9%	67.2%	63.9%

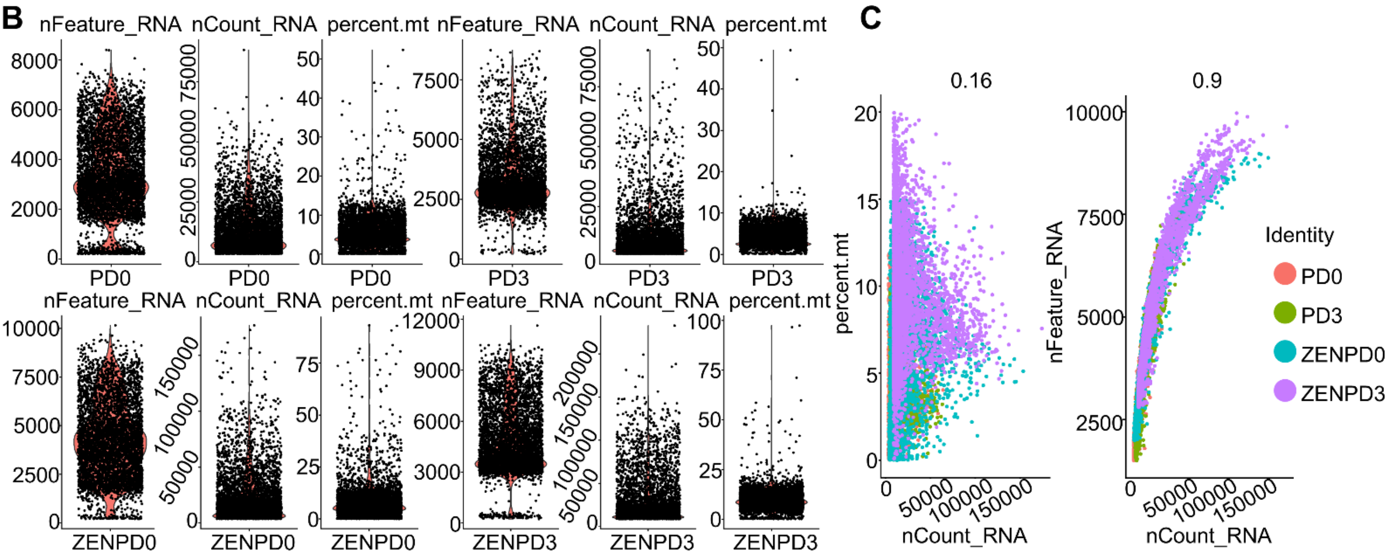

Figure S1. Ovarian single cell sequencing information and quality control results

(A) Sequencing data statistics of four sets of single cell samples. (B) Violin plot demonstrating the number of genes (nFeature_RNA), unique molecular identifier (nCount_RNA), and percentage of mitochondrial genes (percent.mt) in the different data sets. (C) The scatter plot shows the correlation between nCount_RNA, nFeature_RNA, and percent.mt.

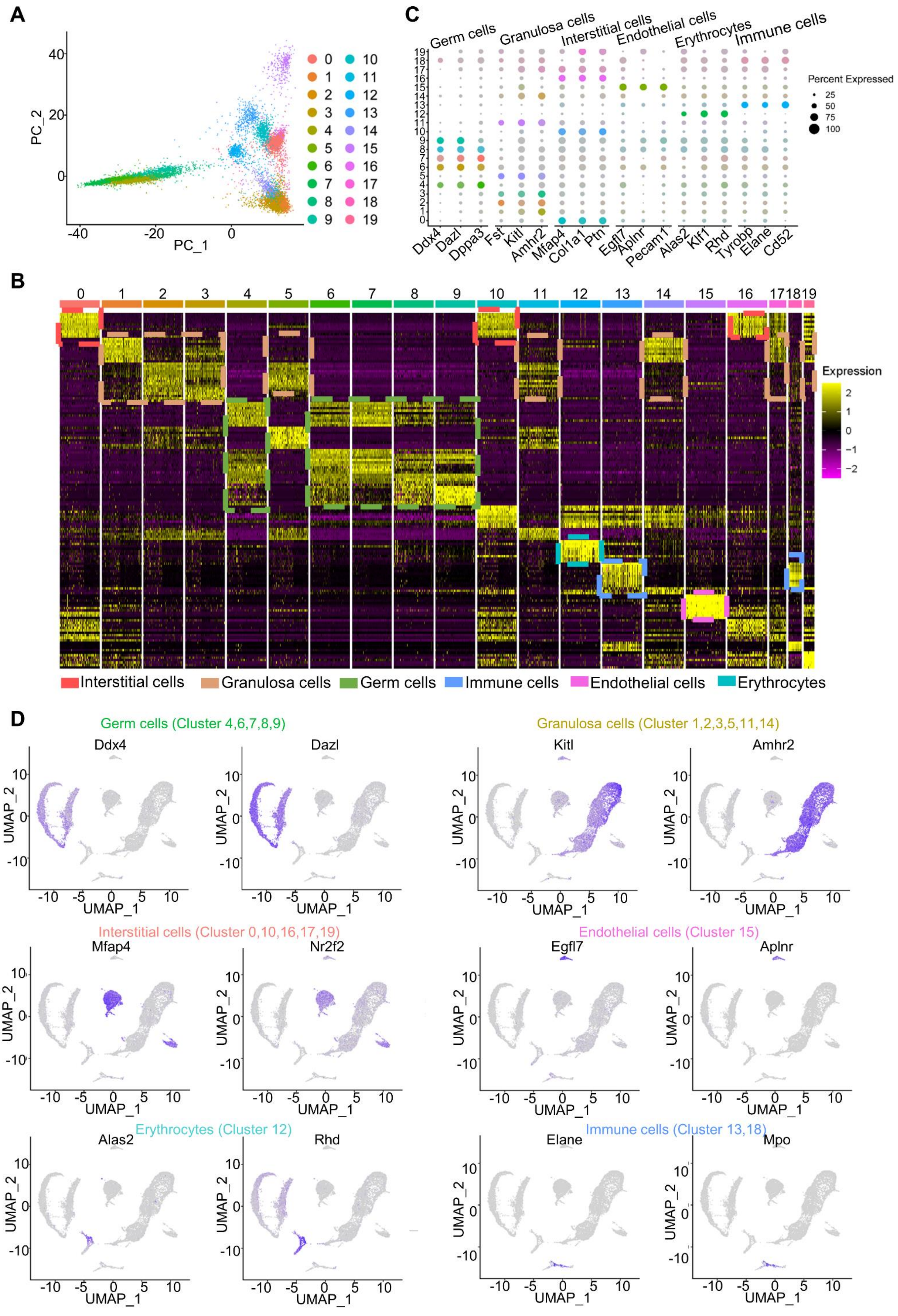
A
PC_1
PC_2
B
Expression
Interstitial cells
Granulosa cells
Germ cells
Immune cells
Endothelial cells
Erythrocytes
C
Germ cells
Granulosa cells
Interstitial cells
Endothelial cells
Erythrocytes
Immune cells
Percent Expressed
Ddx4
Dazl
Dppa3
Fst
Kitl
Amhr2
Mfap4
Col1a1
Ptn
Egfl7
Aplnr
Pecam1
Alas2
Klf1
Rhd
Tyrobp
Elane
Cd52
D
Germ cells (Cluster 4,6,7,8,9)
Granulosa cells (Cluster 1,2,3,5,11,14)
Interstitial cells (Cluster 0,10,16,17,19)
Endothelial cells (Cluster 15)
Erythrocytes (Cluster 12)
Immune cells (Cluster 13,18)
Nr2f2
Mpo
UMAP_1
UMAP_2

Figure S2. Expression of marker genes in each cell cluster

(A) PCA chart shows the position of all cells. (B) The heatmap shows the top 10 DEGs of each cell cluster. (C) The bubble chart shows the expression of genes specifically expressed in different cell clusters. The size of the dot represents the percentage of cells expressing the specified gene in each cluster, and the color intensity of the dot represents the average expression level of the specified gene. (D) UMAP map of the expression level of cell marker genes in each type. Germ cells: *Ddx4* and *Dazl*; Granulosa cells: *Kitl* and *Amhr2*; Interstitial cells: *Mfap4* and *Nr2f2*; Endothelial cells: *Egfl7* and *Aplnr*; Erythrocytes: *Alas2* and *Rhd*; Immune cells: *Elane* and *Mpo*.

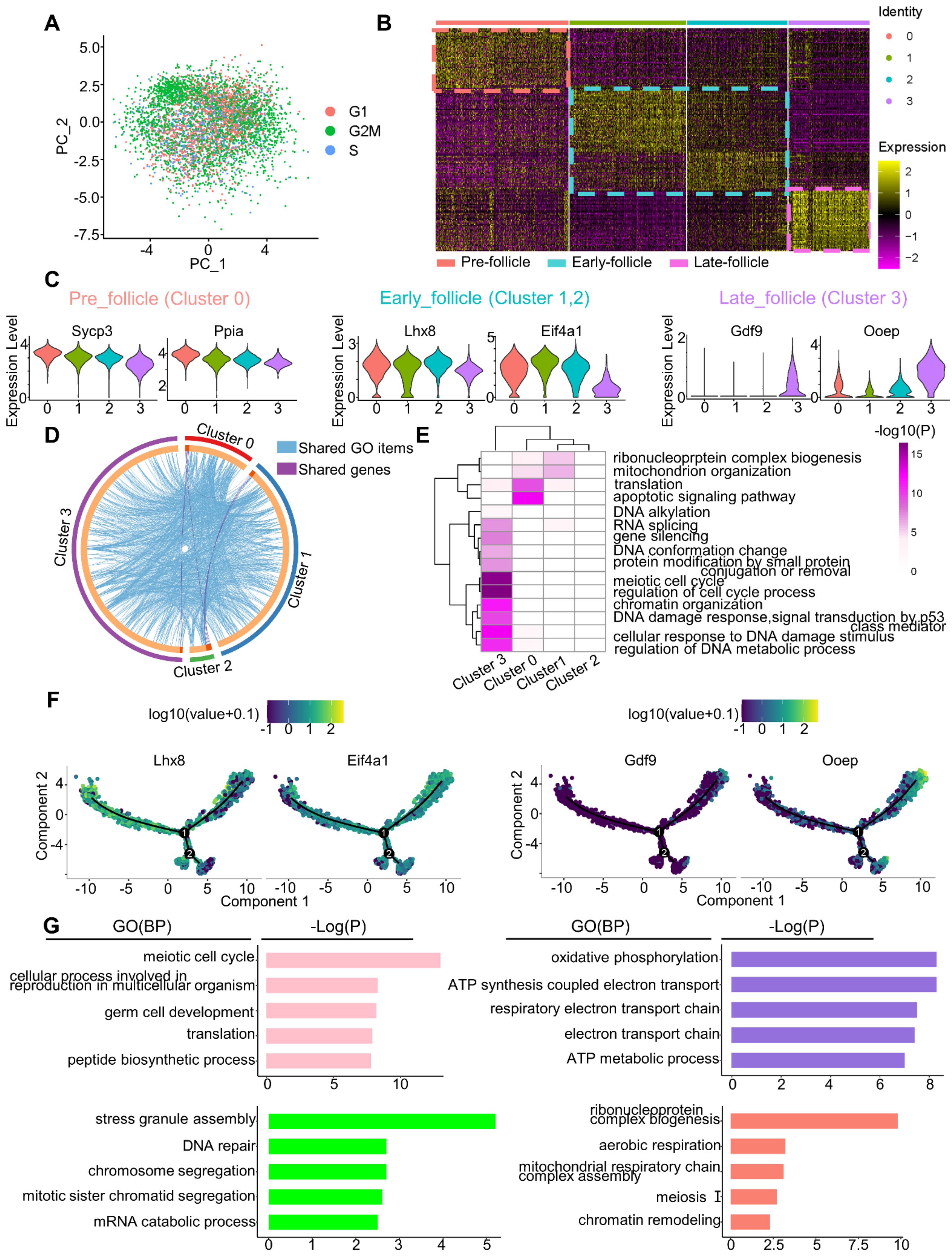
A
PC_2
PC_1
G1
G2M
S
B
Identity
Expression
Pre-follicle
Early-follicle
Late-follicle
C
Pre_follicle (Cluster 0)
Early_follicle (Cluster 1,2)
Late_follicle (Cluster 3)
Expression Level
Sycp3
Ppia
Lhx8
Eif4a1
Gdf9
Ooep
D
Cluster 0
Cluster 1
Cluster 2
Cluster 3
Shared GO items
Shared genes
E
-log10(P)
ribonucleoprptein complex biogenesis
mitochondrion organization
translation
apoptotic signaling pathway
DNA alkylation
RNA splicing
gene silencing
DNA conformation change
protein modification by small protein conjugation or removal
meiotic cell cycle
regulation of cell cycle process
chromatin organization
DNA damage response,signal transduction by p53 class mediator
cellular response to DNA damage stimulus
regulation of DNA metabolic process
Cluster 3
Cluster 0
Cluster1
Cluster 2
F
log10(value+0.1)
Lhx8
Eif4a1
Gdf9
Ooep
Component 2
Component 1
G
GO(BP)
-Log(P)
meiotic cell cycle
cellular process involved in reproduction in multicellular organism
germ cell development
translation
peptide biosynthetic process
oxidative phosphorylation
ATP synthesis coupled electron transport
respiratory electron transport chain
electron transport chain
ATP metabolic process
stress granule assembly
DNA repair
chromosome segregation
mitotic sister chromatid segregation
mRNA catabolic process
ribonucleoprotein complex biogenesis
aerobic respiration
mitochondrial respiratory chain complex assembly
meiosis I
chromatin remodeling

Figure S3. Dissecting germ cell subclusters

(A) PCA diagram shows that after removing the effect of cell cycle from the germ cells, the cells were no longer separated into stages according to G1, G2/M, and S phases. (B) The heatmap shows the top 20 DEGs of each cell cluster. The germ cell clusters are divided into Pre-, Early-, and Late-follicle. (C) Violin chart shows the average expression level of the three stage marker genes in different cell clusters (Pre-follicle: *Sycp3* and *Ppia*; Early_follicle: *Lhx8* and *Eif4a1*; Late_follicle: *Gdf9* and *Ooep*). The plot uses the color of the cell cluster in the UMAP diagram. (D) Circos diagram shows the DEGs and GO terms shared between different germ cell clusters. The purple line represents Shared DEGs, and the blue line represents Shared GO terms. (E) Heatmap shows the GO term enrichment results of four germ cell clusters. (F) Gene expression levels and positions of *Lhx8*, *Eif4a1*, *Gdf9,* and *Ooep* on single-cell pseudotime trajectories. (G) The histogram shows the GO terms enrichment results of four gene sets (top 50).

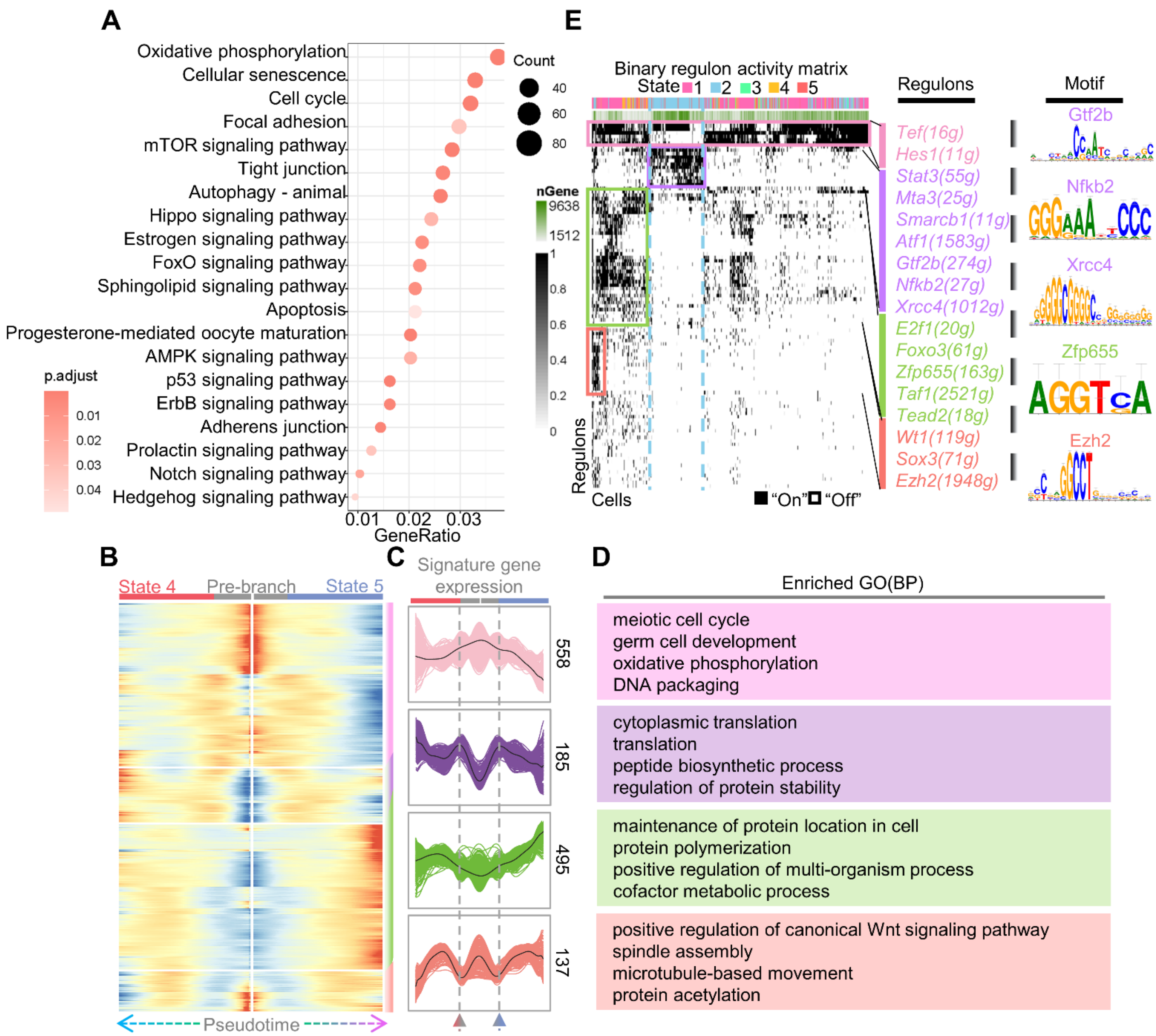

Figure S4. Single-cell pseudotime trajectories of germ cells

(A) KEGG enrichment results of DEGs (top 20). (B) The heatmap shows the changes in gene expression of all germ cells in the two cell fate branches at point 2. (C) The graph shows the expression of the genome in the pseudotime trajectory. (D) GO term enrichment results of four gene sets (top 50). (E) The SCENIC binary regulon activity matrix shows the regulon enrichment of a specific cell state. Each column represents a single cell, and each row

represents a rule. “On” represents an effective regulon, and “Off” represents an ineffective regulon.

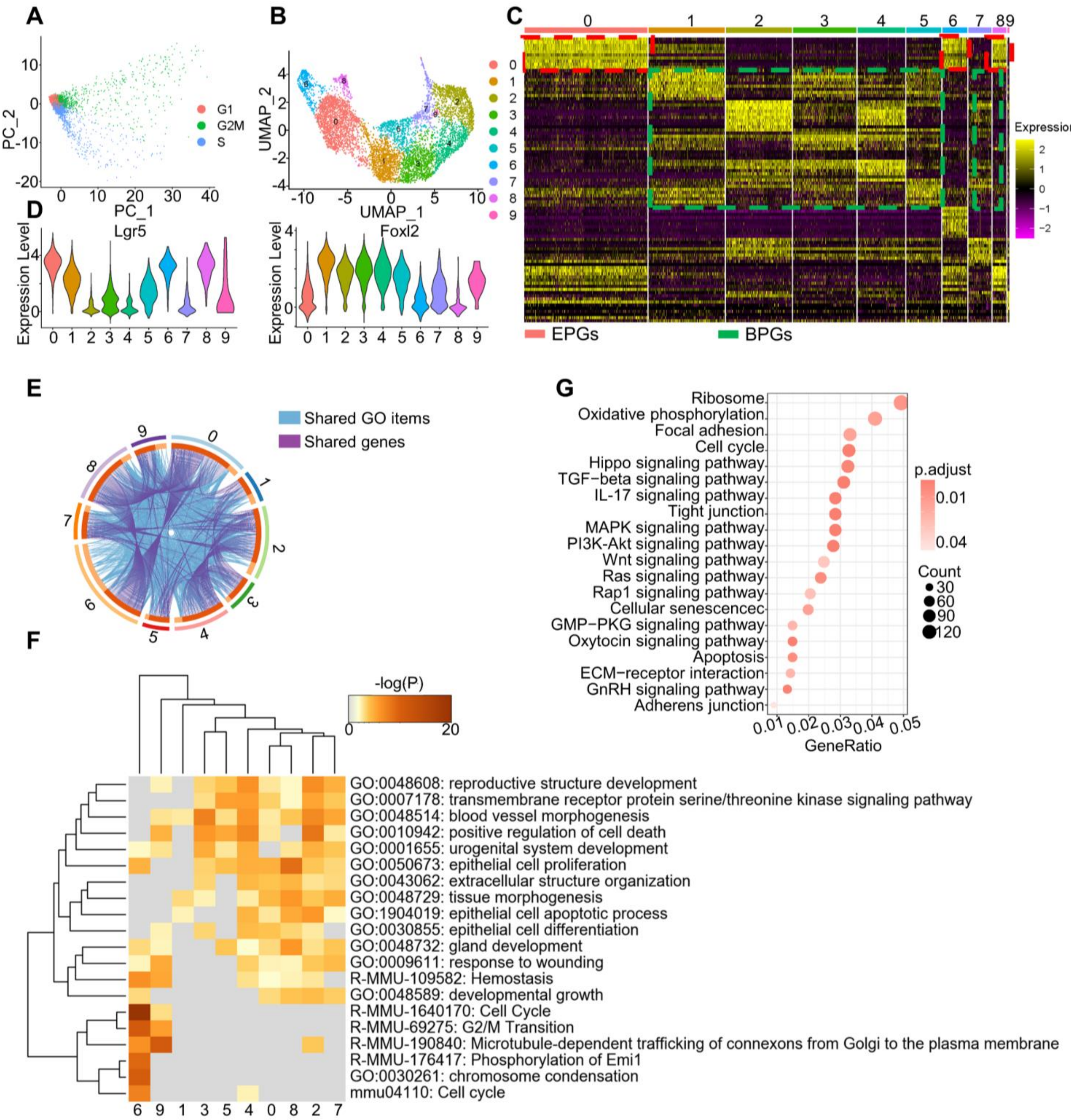

Figure S5. Dissection of granulosa cell subclusters

(A) PCA chart shows that granulosa cells are not separated in stages according to G1, G2/M, and S phases. (B) Subpopulation of granulosa cell clusters. (C) Heatmap shows the top 20

DEGs of each cell cluster. Granulosa cell clusters are divided into EPGs and BPGs. (D) Violin chart shows the average expression levels of the marker genes at the two stages in different cell clusters (EPGs: *Lgr5*; BPGs: *Foxl2*). This plot uses the color of the cell cluster in the UMAP diagram. (E) Circos diagram shows the DEGs and GO terms shared between different granulosa cell clusters. The purple line represents Shared DEGs, and the blue line represents Shared GO terms. (F) Heatmap shows the enrichment results of the granulosa cell clusters. (G) KEGG enrichment results of DEGs (top 20).

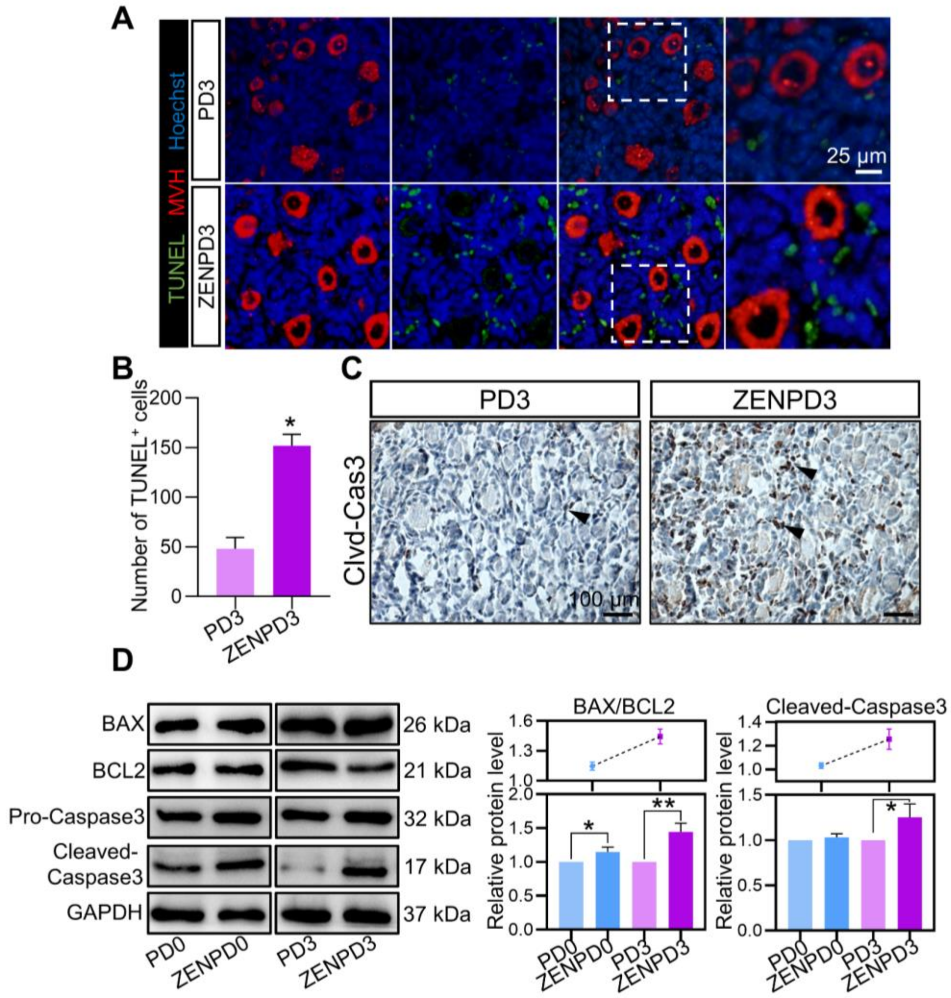

Figure S6. ZEN exposure promoted apoptosis of ovarian granulosa cells

(A) TUNEL staining of PD3 ovary. MVH labeled oocytes (red); TUNEL labeled apoptotic cells (green); nuclei were stained with Hoechst (blue). (B) Quantitative analysis of apoptotic cells. (C) Cleaved-Caspase3 staining of PD3 ovary in the control group and the treatment group. (D) Western blot analysis results of BAX, BCL2, Pro-Caspase3, Cleaved-Caspase3, and GAPDH. The percentage of each group is presented as the mean ± standard deviation. All experiments were repeated at least three times (*$P < 0.05$; **$P < 0.01$).

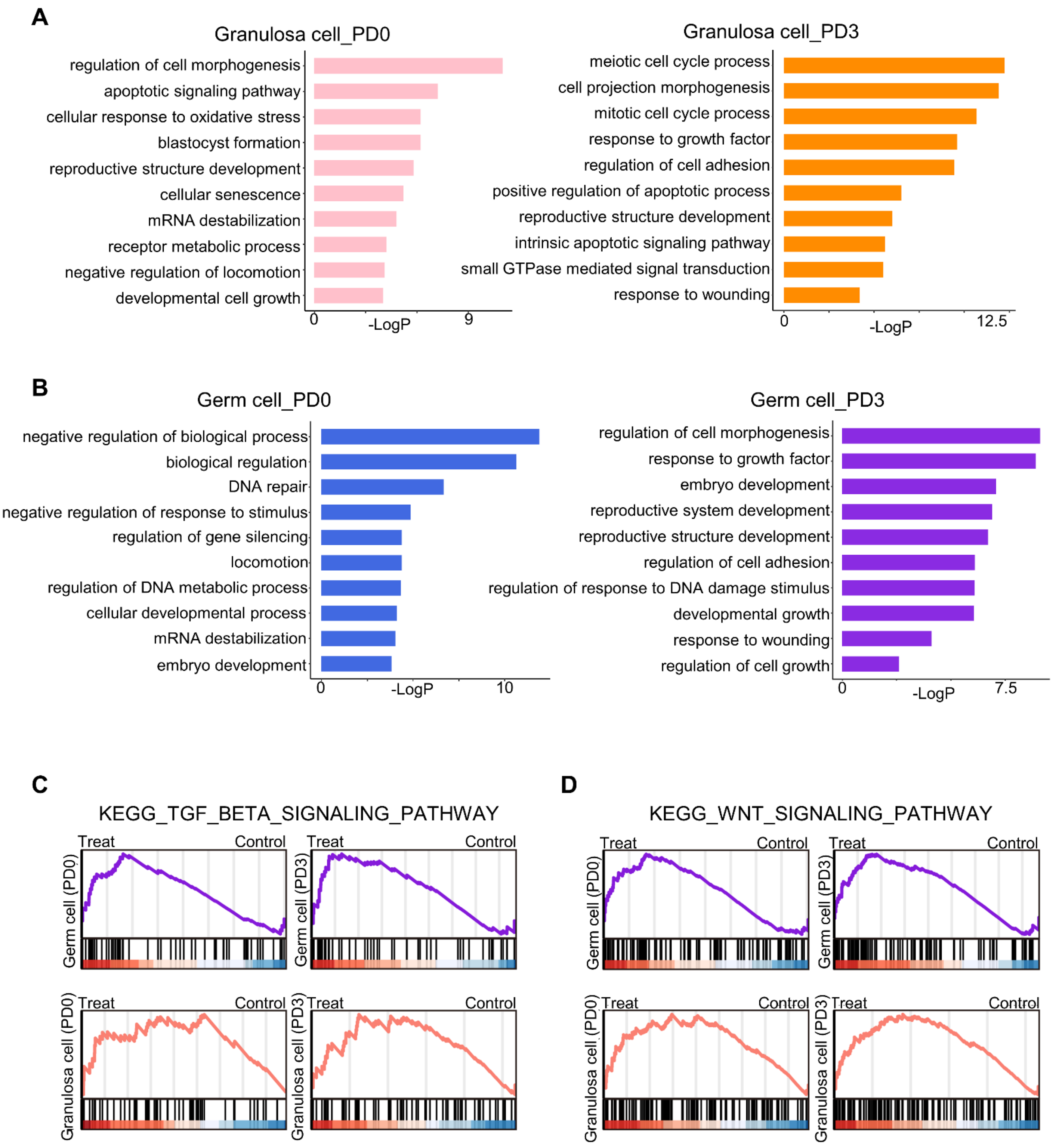

Figure S7. DEGs enrichment analysis

(A) The bar graph shows the results of the GO term enrichment of the DEGs of PD0 and PD3 ovarian granulosa cells. (B) The histogram shows the results of the GO term enrichment of

PD0 and PD3 ovarian germ cell DEGs. (C) GSEA of the TGF-beta signaling pathway. (D) GSEA of the Wnt signaling pathway.

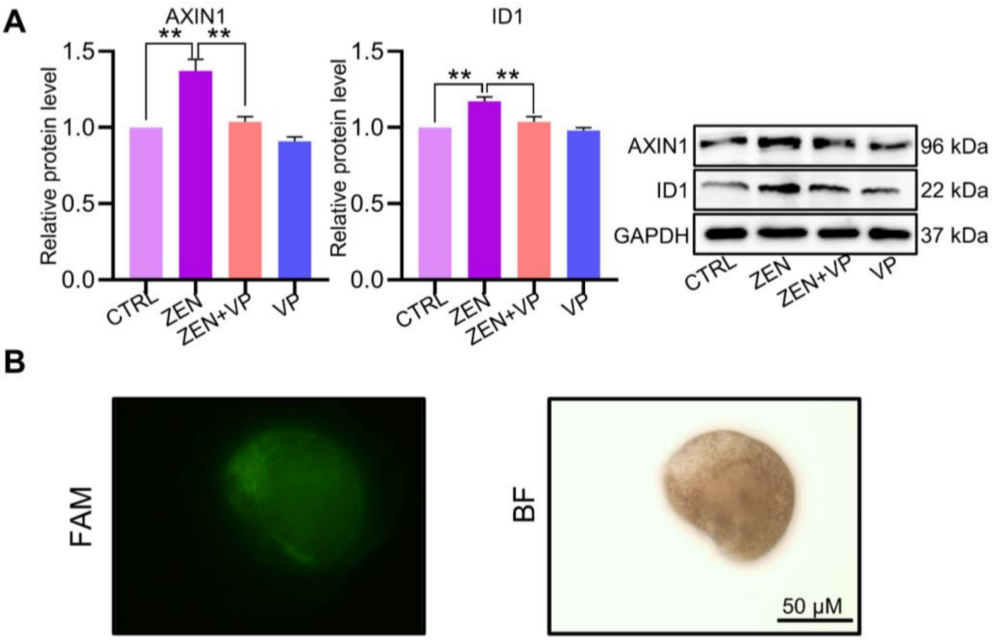

Figure S8. *In vitro* culture of ovaries

(A) Representative Western blot and analysis results of AXIN1, ID1, and GAPDH. (B) Representative image of mouse ovary cultured with the *Yap1* siRNA group. The percentage of each group is presented as the mean ± standard deviation. All experiments were repeated at least three times (**P* < 0.05; ***P* < 0.01).

Supplemental Tables

Table S1. Antibodies used in this paper

**Primary antibodies**	**Vendor**	**Dilution**	**Source**
AXIN1 (WB)	Affinity (DF9264)	1:1000	Rabbit
BMP2 (WB)	Affinity (AF5163)	1:1000	Rabbit
BMPR1B (WB)	Affinity (DF6748)	1:500	Rabbit
BAX (WB)	CST (2772S)	1:1000	Rabbit
BCL2 (WB)	Beyotime (AB112)	1:1000	Rabbit
Caspase3 (IHC/WB)	Abcam (ab13847)	1:200/1:1000	Rabbit
FZD2 (WB)	Affinity (AF5282)	1:5000	Rabbit
GAPDH(WB)	Affinity (T0004)	1:1000	Mouse
ID1 (WB)	Affinity (DF2932)	1:1000	Rabbit
LHX8 (IHC/WB)	Sigma (SAB2101342)	1:200/1:1000	Rabbit
MVH (WB)	Abcam (ab13840)	1:1000	Rabbit
SMAD4 (WB)	Affinity (AF5247)	1:1000	Rabbit
P-SMAD4 (Thr276) (WB)	Affinity (AF8316)	1:500	Rabbit
TEAD1 (WB)	ABclonal (A6768)	1:1000	Rabbit
TEAD2 (WB)	ABclonal (A17407)	1:1000	Rabbit
WNT4 (WB)	Affinity (DF9040)	1:500	Rabbit
YAP1 (IHC/WB)	Affinity (AF6328)	1:100/1:500	Rabbit
P-YAP (S127) (WB)	Affinity (AP0489)	1:500	Rabbit
γ-H2AX (IF/WB)	Bioworld (P16104)	1:200/1:1000	Mouse
**Secondary antibodies**			
CY3-conjugated goat anti-rabbit (IF)	Beyotime (A0516)	1:200	Goat
FITC-conjugated goat anti-rabbit (IF)	Beyotime (A0562)	1:200	Goat
FITC-conjugated goat anti-mouse (IF)	Beyotime (A0568)	1:150	Goat
HRP-conjugated goat anti-Mouse IgG (WB)	Beyotime (A0216)	1:1000	Goat
HRP-conjugated goat anti-rabbit IgG (IHC/WB)	Beyotime (A0258)	1:200/1:1000	Goat

Table S2. log2 (Foldchange) of key genes

Gene symbol	Germ cell_PD0	Germ cell_PD3	Granulosa cell_PD0	Granulosa cell_PD3
Wnt4	1.154	1.229	2.499	2.787
Fzd2			1.565	1.190
Bmp2			0.798	0.733
Bmpr1b	1.270	1.187	0.638	0.585
Smad4	1.602	1.902	1.629	1.981
Yap1	0.639	0.609	0.543	0.599
Tead1	1.175	1.531	1.113	1.598
Tead2		0.370	-0.905	-1.230
Axin1	1.272	1.436	1.433	1.859
Id1	0.580	0.329	0.959	0.818

Table S3. Software and Algorithms

ImageJ	v 1.48
AlphaView SA	3.4.0
Image-Pro Plus	5.1
R-3.6.3	https://www.r-project.org
CellRanger	https://www.10xgenomics.com/
Seurat V3.1	https://satijalab.org/seurat/v3.1/immune_alignment.html
Metascape	http://metascape.org
Cytoscape	https://cytoscape.org/
Monocle 2	http://cole-trapnell-lab.github.io/monocle-release/docs/
SCENIC	https://github.com/aertslab/SCENIC
CellPhoneDB	https://www.cellphonedb.org/

Table S4. Primers Used for Quantitative RT-PCR

Genes	Forward primer sequence	Reverse primer sequence
Gapdh	GTCGGAGTGAACGGATTTGGC	CACCCCATTTGATGTTGGCG
Yap1	ACCCTCGTTTTGCCATGAAC	TGTGCTGGGATTGATATTCCGTA
